# Chemical Composition, Antibacterial Activity, and Antibiotic Potentiation of *Boswellia sacra* Flueck. Oleoresin Extracts from the Dhofar Region of Oman

**DOI:** 10.1155/2021/9918935

**Published:** 2021-05-24

**Authors:** Luay Rashan, Alan White, Manon Haulet, Nicolas Favelin, Parag Das, Ian Edwin Cock

**Affiliations:** ^1^Frankincense and Biodiversity Group, Dhofar University, Salalah 2509, Oman; ^2^School of Environment and Science, Griffith University, 170 Kessels Rd, Nathan, Queensland 4111, Australia; ^3^School of Biology, Ecole de Biologie Industrielle (EBI), Cergy, France; ^4^Oman Pharmaceutical Products Co. LLC, Salalah, Oman; ^5^Environmental Futures Research Institute, Nathan Campus, Griffith University, 170 Kessels Rd, Nathan, Queensland 4111, Australia

## Abstract

The emergence of MDR bacterial pathogens has directed antibiotic discovery research towards alternative therapies and traditional medicines. *Boswellia sacra* oleoresin (frankincense) was used to treat bacterial infections in traditional Arabian and Asian healing systems for at least 1000 years. Despite this, *B. sacra* extracts have not been rigorously tested for inhibitory activity against gastrointestinal pathogens or bacterial triggers of autoimmune diseases. Solvent extracts were prepared from *Boswellia sacra* oleoresins obtained from three regions near Salalah, Oman. MIC values were quantified against gastrointestinal pathogens and bacterial triggers of selected autoimmune diseases by disc diffusion and broth dilution methods. The antibacterial activity was also evaluated in combination with conventional antibiotics, and the class of interaction was determined by ΣFIC analysis. Isobolograms were used to determine the optimal ratios for synergistic combinations. Toxicity was evaluated by ALA and HDF cell viability bioassays. The phytochemical composition of the volatile components of all extracts was identified by nontargeted GC-MS headspace analysis. All methanolic extracts inhibited the growth of all of the bacteria tested, although the extracts prepared using Najdi oleoresin were generally more potent than the Sahli and Houjari extracts. Combinations of the methanolic *B. sacra* extracts and conventional antibiotics were significantly more effective in inhibiting the growth of several bacterial pathogens. In total, there were 38 synergistic and 166 additive combinations. Approximately half of the synergistic combinations contained tetracycline. All *B. sacra* extracts were nontoxic in the ALA and HDF cell viability assays. Nonbiased GC-MS headspace analysis of the methanolic extracts putatively identified a high diversity of monoterpenoids, with particularly high abundances of *α*-pinene. The antibacterial activity and lack of toxicity of the *B. sacra* extracts indicate their potential in the treatment and prevention of gastrointestinal and autoimmune diseases. Furthermore, the extracts potentiated the activity of several conventional antibiotics, indicating that they may contain resistance-modifying compounds.

## 1. Introduction

The World Health Organisation (WHO) considers antibiotic-resistant bacteria to be one of the most urgent issues facing medical science [1]. The development of widespread antibiotic-resistant strains of many common bacterial pathogens in recent years has reduced the efficacy of many clinical antibiotics and in many cases has rendered common clinical antibiotics of little or no use to combat infections. This trend is expected to increase in future years with the transfer of resistance genes between strains of the same bacterial species and between species. Several classes of antibiotics are now of limited efficacy in a growing number of pathogens, which are now classified as multidrug-resistant (MDR) strains. Of considerable concern, some strains are now totally drug resistant (TDR) to all common clinical antibiotics and there are few effective treatment modalities to treat these infections. Indeed, several important human bacterial pathogens including *Klebsiella pneumoniae* and *Neisseria gonorrhoeae* have already been reported to be resistant to all current antibiotic chemotherapies [2]. Furthermore, a TDR strain of *Mycobacterium tuberculosis* has evolved in recent years and is now relatively common in some areas of the world, including Africa and some parts of Asia. There is a lack of effective treatments against that strain, and given the highly infectious nature of *M. tuberculosis* and the high mortality of the active form of the disease, tuberculosis has the potential to cause substantial + loss of life in future years. Indeed, this disease has the potential to cause substantially greater mortality than the current COVID-19 pandemic and new antibiotic therapies against this (and other MDR pathogens) are urgently needed.

For reasons reviewed elsewhere [2], it is unlikely that previous pathways for the development on new antibiotics from microbial sources, or from the modification of existing chemical scaffolds, will provide adequate antibiotic pipelines in the future. New antibiotic sources and methods of antibiotic therapy are urgently required. An examination of traditional plant-based medicine systems for the development of new antibacterial therapeutics is promising as plant-derived medicines have often been used for hundreds (in some cases, thousands) of years and are relatively well documented. Asian, Middle Eastern, and African traditional systems are perhaps the most extensively documented, although many of the therapies are yet to be extensively studied and verified. Frankincense oleoresins are renowned for their therapeutic properties, although they have not been extensively studied to date.

Frankincense oleoresin, obtained from trees of the genus *Boswellia* (family Burseraceae), has long been traded on the Arabian Peninsula and in Northern Africa. Whilst all of the approximately twenty *Boswellia* spp. are used for frankincense production, the oleoresin sourced from *Boswellia sacra* Flueck. is considered to be one of the highest qualities. It is praised for its aromatic and fumigating properties and is widely used in religious practices. It is also valued for its therapeutic effects and has been used in the treatment of gastric disease, dermatitis, and pulmonary disease and for preventing and treating autoimmune diseases (e.g., rheumatoid arthritis) [3]. Several of the therapeutic properties of *B. sacra*, including its analgesic effects and cardioprotective and anti-inflammatory properties, have previously been evaluated and verified [4, 5]. Additionally, the ability of *B. sacra* essential oils to treat urinary tract infections (UTIs) have been verified *in vitro* [6]. Many of these conditions are caused by bacterial or fungal infections, and several studies have reported the growth inhibitory properties of *B. sacra* essential oils against several important bacterial and fungal human pathogens including *Aspergillus* spp., *Candida albicans*, *Escherichia coli*, *Malassezia furfur*, *Propionibacterium acnes*, *Proteus mirabilis, Proteus vulgaris, Pseudomonas aeruginosa,* and *Staphylococcus aureus* [7–10].

The previous studies have examined the antimicrobial activity of essential oils, which would have significantly altered compositions compared to the oleoresins. Those studies have focussed on the volatile mono- and sesquiterpenoid components present in the essential oils. However, many other nonvolatile components would be lost during the distillation process and the essential oils may have substantially different properties to the unprocessed oleoresin. Furthermore, due to the physiochemical properties of essential oils, the MICs obtained in the earlier studies are difficult to compare to those of other plant extracts. Indeed, a recent study reported MIC values up to 55 mg/mL against some pathogens [7]. Notably, these values indicate relatively low activity, possibly due to the loss of antimicrobial or potentiating compounds during the distillation process. We were unable to find studies that tested the therapeutic properties of *B. sacra* extracts, which would contain the nonvolatile and the volatile components. In contrast, extracts prepared from *Boswellia serrata* Roxb. have previously been studied and MIC values as low as 60 *μ*g/mL were reported against some bacterial pathogens [11, 12].

Furthermore, whilst several of the earlier studies have reported noteworthy activity for *Boswellia* spp. oleoresin preparations, all have myopically focussed on the direct antibacterial effects of the oleoresin products themselves and have neglected to determine whether *Boswellia* spp. preparations can potentiate the activity of other antibacterial preparations and/or antibiotic compounds. This is particularly surprising as frankincense is often used in combination with other plant materials, especially *Commiphora myrrha* (Nees) Engl. (commonly known as myrrh) oleoresin [12] or in combination with conventional antimicrobial therapies [2]. It is therefore important to determine if *Boswellia* spp. preparations have combinatorial effects (particularly potentiating or antagonistic effects) when used with other therapies. The current study examined the ability for *B. sacra* extracts to inhibit the growth of panels of human gastrointestinal bacterial pathogens, and autoimmune disease-stimulating bacteria, both alone and in combination with selected conventional antibiotics.

## 2. Materials and Methods

### 2.1. *Boswellia sacra* Oleoresin Source, Harvesting, and Quality

**Figure 1:**
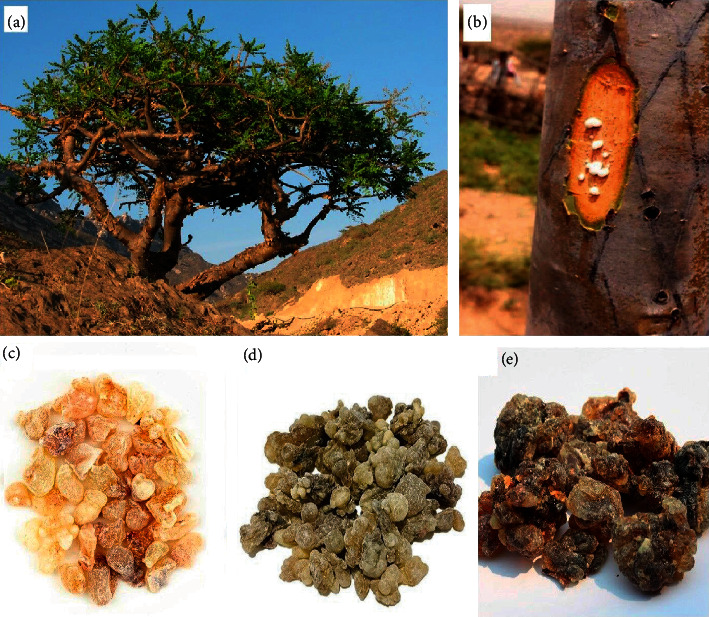
(a) A *Boswellia sacra* tree in the Dhofar region near Salalah; (b) scarification of a *B. sacra* tree trunk and formation of high-quality oleoresin; (c) first-grade (Houjari) oleoresin; (d) second-grade (Sahli) oleoresin; and (e) third-grade (Najdi) oleoresin.

Oleoresins were collected from verified *Boswellia sacra* Flueck. trees (Figure 1(a)) during 2016 from various sites in the Dhofar region of Oman, within 100 km of the centre of Salalah (Table 1). The oleoresins were produced by scarification (Figure 1(b)) and collected by traditional methods. The Najdi samples were collected from verified trees at Wadi Doka, on the plateau region north of Salalah. This region experiences a desert climate, with low rainfall (<100 mm annually) and sharp temperature variations throughout the day. The samples designated as Sahli were sourced from Al Magseel in the Shabi valley region to the west of Salalah. This region also experiences desert climate, with high temperature and low rainfall (110–360 mm per year), although with higher annual precipitation than that of the Wadi Doka region. The sample called Houjari was sourced from Jebel Samhan, approximately 86 km east of Salalah. These trees grow at higher altitudes in cooler temperatures than either the Najdi or Sahli cultivars. This region also has substantially higher rainfall (generally 600–700 mm per year).

Three different oleoresins of different grades were obtained from different regions of Dhofar, near Salalah. The grade 1 (also known as Houjari) oleoresin (Figure 1(c)) is the lightest in colour and has the largest clump size. Grade 1 oleoresin is considered the highest quality and is substantially more expense than the other grades. The second-grade (also known as Sahli) oleoresin (Figure 1(d)) is pale yellow to brown in colour, whilst the third-grade (also known as Najdi) oleoresin (Figure 1(e)) is darker in colour, is the cheapest, and is generally considered to be lower quality than the other grades. The oleoresins were authenticated by comparison with preserved voucher samples stored at the Herbarium, Nizwa University, Oman. The samples were transported to Australia for phytochemical and bioactivity investigation, and voucher specimens (Table 1) are stored at Griffith University, Australia.

### 2.2. Extraction of *B. sacra* Oleoresins

Prior to extraction, the individual oleoresins were ground to a coarse powder using a mortar and pestle. Individual 1 g masses were weighed into separate tubes, and 50 mL volumes of AR grade methanol (Ajax Fine Chemicals, Australia) or sterile deionised water was added; and the ground oleoresin was extracted by maceration at three different temperatures:

- Extraction method 1: the oleoresin was extracted for 24 hours at 4°C with gentle shaking.
- Extraction method 2: the oleoresin was extracted for 24 hours at 23°C with gentle shaking.
- Extraction method 3: the oleoresin was extracted for 24 hours at 35°C with gentle shaking.

The resultant extracts were filtered through filter paper (Whatman No. 54) under vacuum and dried in a vacuum oven at 35°C until there was no further change in mass following additional drying time. The dried extracts were weighed, the extraction yield was calculated, and the extracts were redissolved in 10 mL deionised water (containing 1% DMSO).

### 2.3. Qualitative Phytochemical Studies

Qualitative analysis of the chemical composition of the extracts for the presence of alkaloids, anthraquinones, cardiac glycosides, flavonoids, phenolic compounds, phytosterols, saponins, tannins, and triterpenoids was conducted by previously described assays [13–15].

### 2.4. Antibacterial Screening

#### 2.4.1. Test Bacteria

*Acinetobacter baylyi* (ATCC33304), *Escherichia coli* (ATCC O157 H7), *Klebsiella pneumoniae* (ATCC31488), *Proteus mirabilis* (ATCC21721), *Shigella sonnei* (ATCC 25931), *Staphylococcus aureus* (ATCC 157293), and *Pseudomonas aeruginosa* (ATCC39324) were purchased from the American Type Culture Collection (ATCC), USA. The clinical bacterial strains of *Enterococcus faecalis* and *Salmonella newport* were obtained from the School of Environment and Science teaching laboratory at Griffith University. All bacteria were subcultured and maintained aerobically in Mueller-Hinton broth and on Mueller-Hinton agar at 37°C. All media powders were obtained from Oxoid Ltd., Australia. The antibacterial screening studies conformed to CLSI standardised methods [16].

#### 2.4.2. Standard Antibiotics

All standard antibiotic controls used in the microplate liquid dilution assays were purchased from Sigma-Aldrich (Australia) as powders at the following purities/potencies: Penicillin-G (potency of 1440–1680 *μ*g/mg), chloramphenicol (≥98% purity by HPLC), erythromycin (potency of ≥850 *μ*g/mg), ciprofloxacin (≥98% purity by HPLC), and tetracycline (≥95% purity by HPLC). All powders were resuspended individually in sterile deionised water and diluted to 10 *μ*g/mL for use in the assay. Preloaded discs of ampicillin (10 *μ*g) and chloramphenicol (10 *μ*g) were obtained from Oxoid Ltd. (Australia) and served as positive controls for the disc diffusion assay.

#### 2.4.3. Evaluation of Antimicrobial Activity

Initial screening of the antimicrobial activity of the oleoresin extracts was achieved by standard disc diffusion assay [17]. Briefly, a 100 *μ*L volume of individual exponential growth-phase bacterial cultures grown in Mueller-Hinton broth (approximately 10^8^ cells/mL) was spread uniformly onto individual Mueller-Hinton agar plates. Susceptibility of the bacteria to growth inhibition by the oleoresin extracts was evaluated using 6 mm sterilised filter paper discs infused with 10 *μ*L of the test extract. All plates were incubated for 24 hours at 37°C. The diameters of the zones of inhibition (ZOIs) were subsequently measured to the closest whole millimetre. All susceptibility assays were performed three times, each with internal triplicates (*n* = 9) and the mean values are reported herein. Standard discs of ampicillin (10 *μ*g) and chloramphenicol (10 *μ*g) were included for each assay as positive controls, whilst discs infused with 10 *μ*L of sterile distilled water were included as negative controls.

#### 2.4.4. Minimum Inhibitory Concentration (MIC) Determination

Two methods were used to determine the minimum inhibitory concentration of each oleoresin extract. Colorimetric broth dilution MIC (checkerboard) assays are sensitive measures of bacterial growth inhibitory activity. They are commonly used to quantify bacterial growth inhibition efficacy, allowing for easy comparison with other studies. Broth dilution MIC (checkerboard) assays were performed by standard methods [18]. A standard solid-phase agar disc diffusion assay using Mueller-Hinton agar plates was also used in this study for comparison and as a closer representation of solid-phase infections.

#### 2.4.5. Broth Microdilution MIC Assay

The MICs of the *B. sacra* oleoresin extracts were determined by standard broth microdilution assays in 96-well microtitre plates [18, 19]. Briefly, 100 *μ*L of the test extracts or control antibiotics (100 *μ*L) was added to individual wells of a 96-well microtitre plate containing 100 *μ*L of sterile broth. A growth control (without extract) and a sterile control (without inoculum) were included on each plate. A further 100 *μ*L of 0.5 McFarland bacterial cultures was added to all wells except the sterile control wells and incubated at 37°C for 24 h. Aliquots (40 *μ*L) of *p*-iodonitrotetrazolium violet (INT; Sigma, Australia) at a concentration of 0.2 mg/mL in sterile deionised water were added into all wells, and the plates were incubated for a further 6 h at 37°C. The MIC was visually determined as the lowest dose at which colour development was inhibited.

#### 2.4.6. Disc Diffusion MIC Quantification

Standard disc diffusion assays were also used to quantify the minimum inhibitory concentration (MIC) of the oleoresin extracts across a range of doses [20]. Graphs of the ZOI versus the Ln of the extract concentration were used to calculate the MIC values of each extract.

### 2.5. Evaluation of Combinational Effects: ΣFIC Assessment

Interactions between the *B. sacra* oleoresin extracts and conventional antibiotics were evaluated via the sum of fractional inhibitory concentrations (∑FIC) method [18]. In order to conduct these experiments, only oleoresin extracts that had appreciable activities (MIC<2000 *μ*g/mL) were tested in combination with antibiotics whose MIC values could be determined against the bacterial strains. The FIC values for each component (*a* and *b*) were calculated using the following equations where *a* represents the plant extract sample and *b* represents the conventional antibiotic:

  FIC(a) = MIC (*a* in combination with *b*)/MIC (*a* independently).
  FIC(b) = MIC (*b* in combination with *a*)/MIC (*b* independently).

The ΣFIC was then calculated using the formula ΣFIC = FIC(a) + FIC(b). The interactions were classified as synergistic (ΣFIC ≤ 0.5), additive (ΣFIC > 0.5–1.0), indifferent (ΣFIC > 1.0–4.0), or antagonistic (ΣFIC > 4.0) [18].

### 2.6. Toxicity Screening

The toxicity of the oleoresin extracts was evaluated by two methods. The *Artemia* nauplii lethality assay (ALA) provided a rapid preliminary evaluation of toxicity, whilst MTS-based cellular viability assays were used to evaluate cytotoxicity towards human cells.

#### 2.6.1. *Artemia franciscana* Nauplii Toxicity Screening

Toxicity was tested using standard *A. franciscana* nauplii lethality assays (ALA) [21, 22]. Briefly, 400 *μ*L of individual extract dilutions or the reference toxin (1000 *μ*g/mL potassium dichromate) was added to 400 *μ*L of artificial seawater containing approximately 50 newly hatched *A. franciscana* nauplii (<1 day from hatching) and incubated at 25°C. Artificial seawater (400 *μ*L) was also included on each plate in triplicate as a negative control. All treatments and controls were performed three times, each with three internal replicates (*n* = 9). Following 24-h exposure, the nauplii were sacrificed by the addition of a drop of glacial acetic acid to each well, the dead nauplii were counted, and the % mortality per well was calculated. The LC_50_ with 95% confidence limits for each treatment was calculated using probit analysis. Oleoresin extracts with LC_50_ > 1000 *μ*g/mL were considered to be nontoxic.

#### 2.6.2. Cell Viability Screening

The toxicity of the oleoresin extracts was also evaluated using normal human primary dermal fibroblasts (HDF) obtained from American Type Culture Collection (ATCC PCS-201-012). All *B. sacra* oleoresin extracts were tested for cytotoxicity towards these cells at 200 *μ*g/mL after 24-h exposure using standard protocols [23]. Three biological repeats, each with three technical replicates (*n* = 9), were tested for each oleoresin extract. The absorbances of each test were recorded using a Molecular Devices Spectra Max M3 plate reader at 540 nm (blank wavelength 690 nm). The % cellular viability of each oleoresin extract was subsequently calculated using the following formula:

(1) $$\begin{array}{c} \text{\% cellular viability}=\frac{\text{Abs test sample}-\left(\text{mean Abs control}-\text{mean Abs blank}\right)}{\left(\text{mean Abs control}-\text{mean Abs blank}\right)}. \end{array}$$

Cellular viabilities >50% (compared to the untreated control viability) were deemed to be nontoxic, whereas tests that resulted in ≤50% of the untreated control viability were classified as toxic.

### 2.7. Therapeutic Index Evaluation

The therapeutic index (TI) of the *B. sacra* oleoresin extracts were calculated to determine their suitability as potential therapeutic agents using the formula:

(2) $$\begin{array}{c} \text{Therapeutic index}=\frac{\text{ALA }{\text{LC}}_{50}}{\text{MIC}}. \end{array}$$

### 2.8. Nontargeted GC-MS Head Space Analysis

Chromatographic separation of the individual oleoresin extracts was achieved using a Shimadzu GC-2010 plus (USA) linked to a Shimadzu MS TQ8040 (USA) mass selective detector [24]. The system was equipped with a Shimadzu AOC-5000 autosampler fitted with a solid-phase microextraction fibre (SPME) handling system equipped with Supelco (USA) divinyl benzene/carbowax/polydimethylsiloxane (DVB/CAR/PDMS) fibres. Chromatographic separation was achieved using a 5% phenyl, 95% dimethylpolysiloxane capillary column (30 m × 0.25 mm × 0.25 *μ*m internal diameter; Restek, USA). Helium was used as the carrier gas at a flow rate of 0.79 mL/min at an injector temperature of 230°C. The SPME was exposed to the sample for 10 m to allow the sample compounds to absorb and then desorbed in the injection port at 250°C for 1 min. The column temperature was initially maintained at 30°C for 2 min, increased to 140°C for 5 min, increased further to 270°C over 5 min, and then maintained at that temperature for the duration of the analysis. The GC-MS interface was maintained at 200°C, and no signal was acquired for 1 min after injection. The mass spectrometer was operated in electron ionisation mode at 70 eV, and analytes were recorded in total ion count (TIC) mode for 45 min across a mass range of 45–450 m/z. Compounds were putatively identified by comparison of mass spectral data with the ChemSpider database.

### 2.9. Statistical Analysis

Data are expressed as mean ± SEM of three independent experiments, each with three internal/technical replicates (*n* = 9). One-way ANOVA followed by Tukey's post hoc analysis was used to calculate statistical significance between control and treated groups (*p* < 0.01 was deemed to be statistically significant).

## 3. Results

### 3.1. Liquid Extraction Yields and Qualitative Phytochemical Screening

Extraction of 1 g of each of the *B. sacra* oleoresins with methanol or deionised water yielded dried extracts ranging from 276 mg (aqueous Houjari extraction method 1 extract) to 687 mg (methanolic Najdi extraction method 1 extract) (Table 2). Methanol was a better solvent than water against all oleoresins, with substantially higher masses of extracted material measured. Qualitative phytochemical studies showed that both methanol and water extracted similar classes and relative amounts of phytochemicals. Similarly, only minor differences in phytochemical profiles were noted between the three *B. sacra* cultivars and between the three different extraction methods. All had moderate levels of phenolics, saponins, and flavonoids, as well as low to moderate levels of triterpenoids and phytosterols. All extracts were generally devoid of all other classes of phytochemicals, or they were below the detection threshold.

### 3.2. Antimicrobial Activity of the Oleoresin Extracts

**Figure 2:**
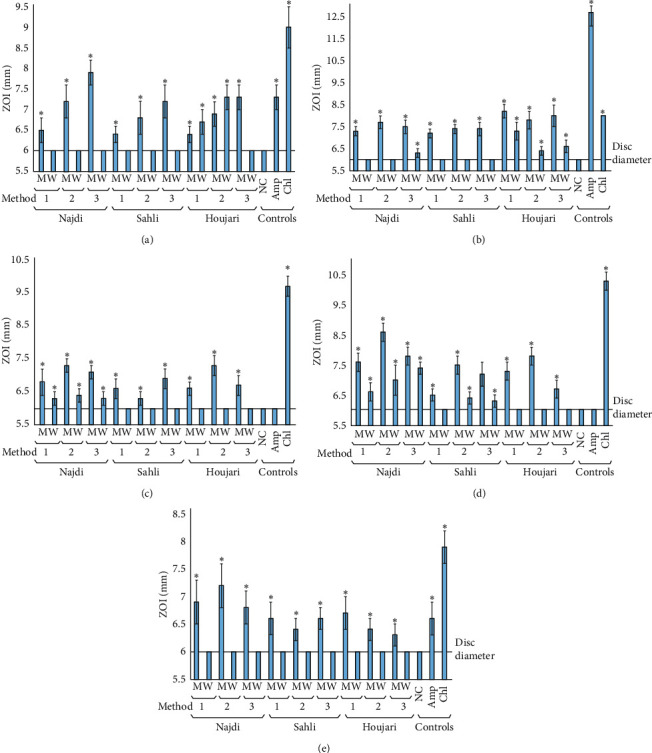
Antibacterial activity of the *B. sacra* oleoresin extracts against gastrointestinal bacterial pathogens measured as zones of inhibition (mm): (a) *E. coli*, (b) *E. faecalis*, (c) *S. aureus*, (d) *S. newport*, and (e) *S. sonnei*. Method refers to the extraction method. *M* = methanolic extract; *W* = water extract; NC = 0.5% DMSO; Amp = ampicillin (10 *μ*g) control; Chl = chloramphenicol (10 *μ*g) control. Results are expressed as mean zones of inhibition ± SEM. The symbol *∗* indicates results significantly different to the untreated control (*p* < 0.01).

To determine the growth inhibitory activity of the *B. sacra* oleoresin extracts against selected human bacterial pathogens, 10 *μ*L volumes were individually applied to filter paper discs and applied to Mueller-Hinton agar plates spread with individual bacteria for testing in the disc diffusion assay. The gastrointestinal bacterial pathogens were inhibited by oleoresin extracts prepared from all *B. sacra* oleoresins and extraction methods (Figure 2). The methanolic extract were substantially better inhibitors of growth of all of the bacteria species tested. Indeed, all aqueous extracts were generally inactive or of only low activity against *E. faecalis*, *S. aureus,* and *S. sonnei*. In contrast, the aqueous Houjari (extraction methods 1 and 2) displayed better inhibition of *E. coli* growth compared with the corresponding methanolic extracts. The Najdi and Sahli (all extraction methods) also inhibited the growth of *S. aureus* and *S. newport*, albeit with much smaller ZOIs measured than for the corresponding methanolic extracts. These inhibitory activities are noteworthy compared with the activity of the antibiotic controls. Indeed, *S. aureus* and *S. newport* were found to be totally resistant to the ampicillin control. Similarly, relatively small ZOIs were measured for ampicillin against *E. coli* and *S. sonnei*, indicating that these extracts may be particularly useful in the treatment of *β*-lactam-resistant gastrointestinal infections. However, testing against other bacterial species and further strains of the bacteria tested in this study is warranted. Notably, *E. faecalis* also displayed low susceptibility to the chloramphenicol control (on the basis of the small ZOI), indicating that this bacterium was also resistant to chloramphenicol. Thus, the methanolic *B. sacra* oleoresin extracts may be particularly useful against both antibiotic-sensitive and antibiotic-resistant gastrointestinal illness, and further studies to screen against bacteria with wider antibiotic resistance spectrums is warranted.

**Figure 3:**
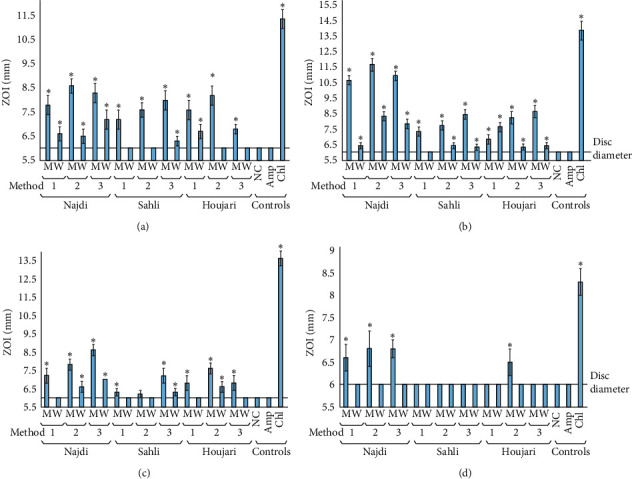
Antibacterial activity of the *B. sacra* oleoresin extracts against bacterial triggers of autoimmune inflammatory diseases measured as zones of inhibition (mm): (a) *P. mirabilis*, (b) *K. pneumoniae*, (c) *A. baylyi*, and (d) *P. aeruginosa*. Method refers to the extraction method; *M* = methanolic extract; *W* = water extract; NC = 0.5% DMSO; Amp = ampicillin (10 *μ*g) control; Chl = chloramphenicol (10 *μ*g) control. Results are expressed as mean zones of inhibition ± SEM. The symbol *∗* indicates results significantly different to the untreated control (*p* < 0.01).

The *B. sacra* oleoresin extracts were also screened against some bacterial pathogens that can trigger specific autoimmune inflammatory diseases in genetically susceptible people (Figure 3). The bacterial species *P. mirabilis* and *K. pneumonia* were selected as they can trigger rheumatoid arthritis and ankylosing spondylitis, respectively, whilst *A. baylyi* and *P. aeruginosa* can both trigger multiple sclerosis [25]. The methanolic extracts of all three oleoresins, produced by all three extraction methods, inhibited the growth of *P. mirabilis* (Figure 3(a)) and *K. pneumonia* (Figure 3(b)) with ZOIs ∼11.5 mm noted against *K. pneumonia*. These activities were particularly noteworthy when compared against the inhibition by the ampicillin control. Indeed, both of these bacteria were resistant to ampicillin, with no inhibition at all evident. In contrast, both bacteria were particularly susceptible to chloramphenicol, with ZOIs in the range of 11.5–14.5 mm. All *B. sacra* aqueous extracts also inhibited the growth of both *P. mirabilis* and *K. pneumonia,* albeit to a substantially lesser extent than noted for the corresponding methanolic extracts. A further trend was evident against these bacteria: The Najdi cultivar generally had slightly stronger growth inhibitory activity against *P. mirabilis* and *K. pneumonia* than the Sahli and Houjari cultivars. Additionally, the extracts prepared using the second extraction method generally had greater activity than the extracts produced using the first or third extraction method. Therefore, these extracts (particularly the methanolic extracts prepared from Najdi oleoresin) have potential in the prevention and treatment of rheumatoid arthritis and ankylosing spondylitis in genetically susceptible people.

Lower activity was noted for the extracts screened against *A. baylyi* (Figure 3(c)) and *P. aeruginosa* (Figure 3(d). Indeed, only the methanolic Najdi extracts (all extraction methods) and the Houjari (extraction method 2) methanolic extract inhibited *P. aeruginosa* growth. All other extracts were completely ineffective against this bacterium. Even for the extracts that displayed inhibitory activity, the small ZOIs (6.5–7.0 mm) are indicative of only low potency. However, it is noteworthy that this bacterium is a particularly antibiotic-resistant strain, with no inhibition noted for ampicillin and only relatively small ZOIs measured for chloramphenicol. These results are consistent with other studies that have also reported that this bacterial strain is resistant to penicillin, nystatin [26], erythromycin, ciprofloxacin [27], ampicillin, and chloramphenicol [19, 26, 28]. Due to the resistance of this bacterium to conventional antibiotics, the methanolic *B. sacra* oleoresin Najdi extracts may still have potential against infections of this bacterium.

Whilst a greater number of the *B. sacra* oleoresin extracts inhibited the growth of the *A. baylyi* strain tested (Figure 3(c)), generally only small ZOIs were measured. The methanolic extracts prepared using extraction methods 2 and 3 (particularly for the Najdi cultivar) were generally stronger inhibitors of *A. baylyi* growth than the other extracts. Notably, this bacterial strain was also resistant to ampicillin, although it was susceptible to chloramphenicol (as determined by ZOIs). In contrast, the aqueous extracts for all oleoresins (prepared by all extraction methods) were ineffective, or were only weak inhibitors of *A. baylyi* growth. However, as the Najdi methanolic extracts (prepared by extraction methods 2 and 3) displayed the best activity against both *A. baylyi* and *P. aeruginosa*, further studies are warranted to identify potential drug leads for the prevention of multiple sclerosis and its treatment once the disease has been triggered.

#### 3.2.1. Quantification of Minimum Inhibitory Concentration (MIC)

The MIC values for each extract were evaluated by two methods (disc diffusion and broth dilution assays) against the bacterial species that showed susceptibility in the disc diffusion assay. Extracts prepared from all oleoresins inhibited the growth of the all bacteria tested, although the methanolic extracts were generally substantially more potent than the aqueous extracts on the basis of the determined MIC values (Table 3). The *S. newport* strain tested in this study was particularly susceptible to the *B. sacra* oleoresin extracts. Indeed, LD MIC values of 146, 86, and 128 *μ*g/mL were determined for all Najdi extracts prepared by methods 1, 2, and 3, respectively. The other oleoresin extracts were also effective against this bacterium, albeit with substantially higher MIC values. These MIC values are especially promising as this bacterium was also resistant to all of the conventional antibiotics tested (MIC values > 1 *μ*g/mL) except ciprofloxacin. As *Salmonella* spp. are major causes of food poisoning and diarrhoea. The *B. sacra* oleoresin extracts tested herein are particularly promising for treating food poisoning, even against resistant infections (especially the methanolic Najdi extracts).

All of the other gastrointestinal pathogens were also susceptible to the methanolic *B. sacra* extracts, although the aqueous extracts were substantially less effective. Indeed, MIC values less than 1000 *μ*g/mL were recorded against all gastrointestinal bacterial strains, although *E. coli* and *S. sonnei* were generally more resistant to the extracts than were the other bacterial species. Interestingly, all of these bacteria were also resistant to all of the conventional antibiotics tested except ciprofloxacin, indicating that all of the methanolic oleoresin extracts may be useful in the treatment of gastrointestinal infections and may provide effective leads in the development of new antibiotic chemotherapies.

The methanolic *B. sacra* oleoresin extracts were similarly effective at inhibiting the growth of the bacterial triggers of selected autoimmune inflammatory diseases. Similar to the trends noted for the gastrointestinal pathogens, the aqueous extracts generally exerted only moderate to low inhibitory activities, whilst noteworthy activity (<1000 *μ*g/mL) was noted for all of the methanolic extracts. Also, similar with the trends for the gastrointestinal pathogens, the extracts prepared from the Najdi cultivar (all extraction methods) were generally more effective than the corresponding extracts prepared from the Sahli or Houjari oleoresins. The *P. mirabilis* and *K. pneumoniae* strains tested in this study were particularly susceptible to the extracts (as judged by MIC values). As these bacteria are causes of rheumatoid arthritis and ankylosing spondylitis, respectively, in genetically susceptible individuals [25], the *B. sacra* extracts tested in our study have potential in the prevention and treatment of these diseases. Of further note, both of these bacteria were multidrug-resistant (MDR) strains, each only being susceptible to two of the five conventional antibiotics tested. Thus, these extracts may prove particularly useful in preventing and treating clinical strains (which are often MDR) of the bacterial triggers of rheumatoid arthritis and ankylosing, although clinical trials are required to verify this.

The effects of the *B. sacra* oleoresin extracts against the bacterial triggers of multiple sclerosis were less definitive. Both *A baylyi* and *P. aeruginosa* can trigger multiple sclerosis in genetically susceptible individuals [25]. Noteworthy growth inhibitory activity was noted for the methanolic *B. sacra* oleoresin extracts. As observed with the other bacteria tested, the Najdi oleoresin extracts were generally substantially more potent than the extracts prepared from the other oleoresins. Notably, the aqueous Najdi extracts (prepared by extraction methods 2 and 3) also had noteworthy activity, with MIC values < 1000 *μ*g/mL. Thus, the Najdi extracts may be useful against one of the bacterial triggers of multiple sclerosis. In contrast, all extracts were substantially less effective against *P. aeruginosa*, which may also trigger multiple sclerosis in genetically susceptible people. Indeed, with the exception of the methanolic Houjari extract prepared using method 2, the MIC values recorded for all extracts indicated only low to moderate activity. However, it is noteworthy that the *P. aeruginosa* strain examined in our study was a particularly resistant strain. Indeed, no inhibition of *P. aeruginosa* growth was noted for penicillin, erythromycin, or tetracycline at any dose tested. Furthermore, whilst chloramphenicol did inhibit *P. aeruginosa* growth, the MIC value 2.5 *μ*g/mL indicated that this bacterium is also resistant to this antibiotic. Only ciprofloxacin was effective at inhibiting the growth of this bacterium. As the *B. sacra* extracts were effective inhibitors of one of the bacterial triggers of multiple sclerosis (*A. baylyi*), they may partially prevent the induction of multiple sclerosis, despite their low efficacy against *P. aeruginosa*.

### 3.3. Assessment of Combinational Effects: ΣFIC Determination

The low MIC values demonstrate that the *B. sacra* extracts have good antibacterial properties against the majority of the bacterial species tested. Thus, these extracts may themselves be useful antibiotic therapies. However, extracts contain multiple components and the activity of crude plant extracts may be substantially stronger than an equivalent amount of the individual antibiotic component(s), indicating that the extracts may contain compounds that synergise the activity of the growth inhibitory components, thereby substantially increasing their potency [2, 29]. Identification of potentiating extracts and individual components (if present) may provide methods to “reactivate” conventional antibiotics, even in bacterial strains resistant to those bacteria. Therefore, a series of experiments were undertaken to determine the effects of combinations of the *B. sacra* oleoresin extracts and conventional antibiotics on the growth of the gastrointestinal and autoimmune panels of bacteria.

#### 3.3.1. Combinational Effects against Gastrointestinal Bacterial Pathogens

A wide range of interactions was evident for combinations of the *B. sacra* oleoresin extracts with conventional antibiotics when tested the panel of gastrointestinal pathogens (Table 4). Only combinations where both the antibiotic and extract components individually inhibited bacterial growth were tested in this study. The majority of the combinations resulted in ΣFIC values > 1.0–4.0 and were therefore classed as noninteractive effects. Indeed, 240 of the 405 combinations (∼59%) were noninteractive against the tested bacteria. Whilst these combinations do not provide additional benefits above those of the individual components alone, they also do not decrease the activity of the other component. This is important information as many people self-prescribe complementary therapies (including frankincense), and it is important that the individual therapies do not negate each other's effects.

Of greater interest, a substantial number of combinations resulted in potentiation of the growth inhibitory activity. Twenty-five (∼6%) of the total combinations showed synergistic effects, with the growth inhibition far stronger than the combined effects of the individual components. Notably, many of these synergistic effects were detected against MDR bacteria using antibiotic components that had only low effects against those species. Thus, the extracts appear to “reactivate” the antibiotic components towards those bacteria, increasing their efficacy substantially. These are noteworthy results and indicate promising new chemotherapies. No synergistic interactions were noted against *E. coli* or *S. sonnei*, whereas six combinations were synergistic against *S. newport* and three each were synergistic against *E. faecalis* and *S. aureus*. Also noteworthy, the extracts only synergised the activity of chloramphenicol, ciprofloxacin, and tetracycline, and therefore, the extract component(s) may be inhibiting a resistance mechanism common to these antibiotics (e.g., efflux pumps). In contrast, no synergistic effects were noted against any gastrointestinal bacterial pathogens in combinations containing penicillin or erythromycin.

A further 119 (∼29%) of the total combinations tested produced additive effects against the gastrointestinal bacteria. Whilst not as strong as the potentiation seen for the synergistic combinations, these are still noteworthy as the combinations enhance the antibiotics efficacy of the treatment. Thus, these combinations would be beneficial in the treatment of gastrointestinal disease caused by these bacteria. In contrast, 21 of the extract/conventional antibiotic combinations (∼5%) resulted in antagonism, i.e., the activity of the combination was decreased substantially compared with the activity of the individual components. Thus, it is recommended that these combinations be avoided when treating gastrointestinal disease caused by these bacteria.

#### 3.3.2. Combinational Effects against Autoimmune Inflammatory Disease-Inducing Bacteria

As noted for the gastrointestinal bacterial panel, the majority of the extract/combinations tested against the bacterial triggers of selected autoimmune diseases resulted in noninteractive effects (Table 4). In total, 167 of the 239 combinations (∼70%) produced noninteractive effects against this panel of bacteria. Thus, these combinations may be safely used without decreasing the efficacy of either component. Notably, three (∼1%) antagonistic combinations were also noted. It is recommended that these combinations be avoided in individuals with these autoimmune diseases. In contrast, 13 (∼6%) synergistic and 47 (∼20%) additive combinations were recorded. Three of the synergistic combinations and several more of the additive combinations were noted against *K. pneumoniae*, which was determined in the MIC studies to be an MDR strain. However, few of these potentiating combinations contained antibiotic components towards, which *K. pneumonia* showed resistance. The majority of the synergistic effects (eight of the thirteen), as well as most of the additive effects, occurred when the combinations were tested against *A. baylyi*, indicating that these combinations may be particularly useful in the prevention and treatment of multiple sclerosis.

### 3.4. Varied Ratio Combination Studies (Isobolograms)

In all cases where synergistic effects were detected, the oleoresin extracts produced using extraction method 2 resulted in more pronounced synergy. Therefore, in the varied ratio studies, where synergy was noted for a conventional antibiotic with oleoresin extracts produced by several extraction methods, only the ratio effects for combinations containing the method 2 oleoresin extracts were examined.

#### 3.4.1. Component Ratios of Synergistic Combinations Containing Najdi Oleoresin Extracts

**Figure 4:**
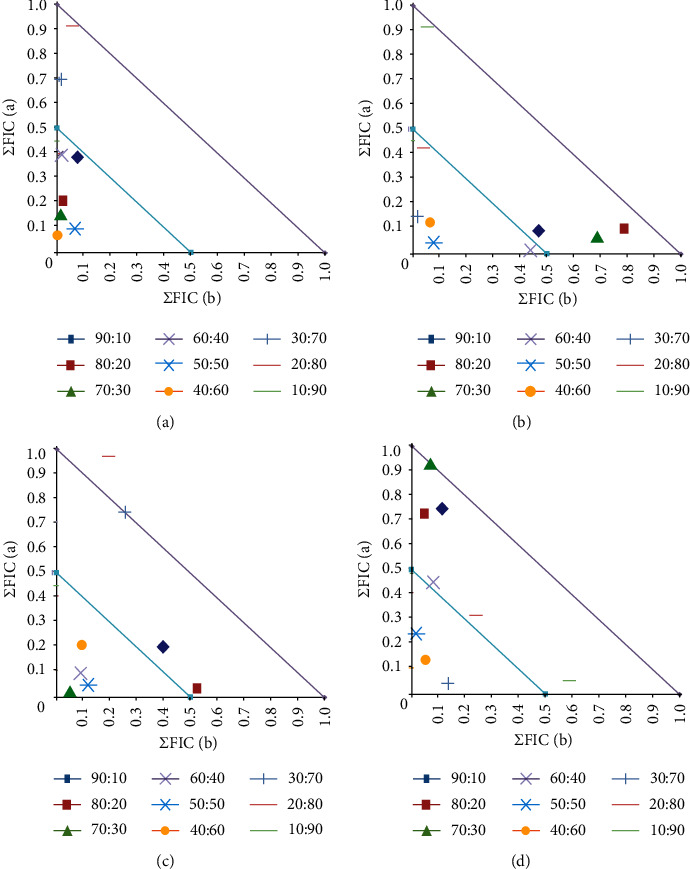
Isobologram for combinations of Najdi (extraction method 2) methanolic extract with chloramphenicol against (a) *S. newport*, (b) ciprofloxacin against *S. aureus*, (c) tetracycline against *K. pneumonia*, and (d) tetracycline against *A. baylyi* extract. The extracts and antibiotics were tested at various ratios against, and results represent mean FIC values of four replicates (*n* = 4). Ratio = % extract:% antibiotic. Ratios lying on or underneath the 0.5 : 0.5 line are considered synergistic (ΣFIC ≤ 0.5). Any points between the 0.5 : 0.5 and 1.0 : 1.0 lines are deemed additive (ΣFIC > 0.5–1.0).

Four combinations of Najdi methanolic extract (produced using extraction method 2) with conventional antibiotics were identified as inducing synergistic interactions (Figure 4). These combinations were further examined using isobologram analysis across a range of extract:antibiotic ratios to identify the ideal ratios to obtain synergy. For the combination of methanolic Najdi extract and chloramphenicol against *S. newport* (Figure 4(a)), the values aligned with the *y* (conventional antibiotic) axis, indicating that chloramphenicol is the major contributor to the antibacterial effect and the extract functions as a potentiator of its activity. Interestingly, only combinations that contained ≥50% extract worked synergistically as growth inhibitors. Thus, these ratio combinations would be beneficial to enhance *S. newport* growth inhibition. However, when used for the treatment of acute gastrointestinal *S. newport* infections, the ratio that maximises the efficacy of the treatment (i.e., the 50 : 50 ratio) would be the preferred option.

In contrast, the values were obtained for the combination of the methanolic Najdi extract (extraction method 2) and ciprofloxacin aligned more closely with the *x* (extract) axis (Figure 4(b)). Thus, whilst ciprofloxacin has substantial activity against *S. aureus*, the combination components whose activity was potentiated are more likely to be present in the extract. In contrast, the results for combinations of the extract and tetracycline against either *K. pneumonia* (Figure 4(c)) or *A. baylyi* (Figure 3(d)) were less definitive. There was not a clear correlation of the measured values with either axis, so it was not possible to determine which component is likely to have its activity increased and which is likely to be the potentiator. Instead, both components in these combinations may potentiate each other's activity. However, we did determine the synergistic ratios in each case. For the *K. pneumonia* isobologram (Figure 4(c)), only combinations containing between 30 and 60% extract induced synergy, with additive effects noted for the other ratios. Thus, any of these ratios would be beneficial in the treatment of *K. pneumonia* infections. Similarly, for the *A. baylyi* isobologram (Figure 4(d)), combinations containing 40 to 60% extract displayed synergy, and thus, these would be the preferred ratios against this bacterium.

#### 3.4.2. Component Ratios of Synergistic Combinations Containing Sahli Oleoresin Extracts

**Figure 5:**
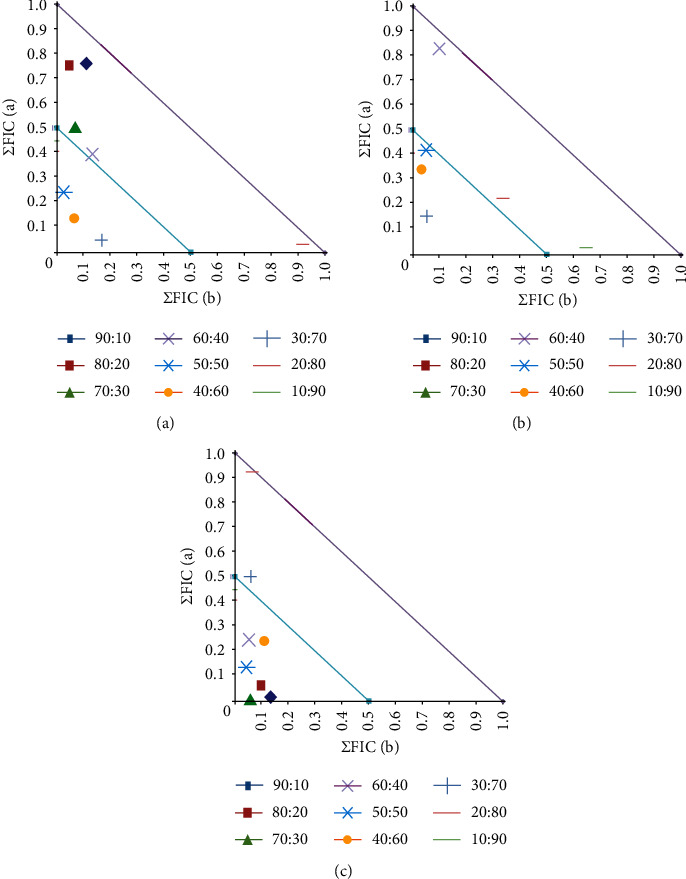
Isobologram for combinations of Sahli (extraction method 2) methanolic extract in combination with tetracycline against (a) *E. faecalis*, (b) *S. newport*, and (c) *A. baylyi*. The extracts and antibiotics were tested at various ratios against, and results represent mean FIC values of four replicates (*n* = 4). Ratio = % extract:% antibiotic. Ratios lying on or underneath the 0.5 : 0.5 line are considered synergistic (ΣFIC ≤ 0.5). Any points between the 0.5 : 0.5 and 1.0 : 1.0 lines are deemed additive (ΣFIC > 0.5–1.0).

Three combinations of the Sahli (extraction method 2) methanolic extract with conventional antibiotics produced synergy and were tested further using various ratios of the extract/antibiotic components (Figure 5). Notably, for all of these isobolograms, the values aligned most closely with the *y* (conventional antibiotic) axis, indicating that in each case the conventional antibiotic contributed most to the antibiotic effect of the combination and the extract potentiated its effect. Of further note, all synergistic combinations containing the Sahli extract also contained that tetracycline, indicating that the extract may block a similar resistance mechanism in each of these bacteria. As tetracycline resistance is most frequently due to the production of tetracycline efflux pumps [30], it is likely that the Sahli contains compounds that function as tetracycline efflux pump inhibitors. For the combinations of the methanolic Sahli extract and tetracycline against *E. faecalis* (Figure 5(a)) and *S. newport* (Figure 5(b)), combinations that contained 30–50% extract were synergistic. Thus, these combination ratios would be beneficial to enhance growth inhibition against *E. faecalis* and *S. newport*. In contrast, combinations containing ≥40% of the extract component (and 10–60% tetracycline) were synergistic against *A. baylyi.*

#### 3.4.3. Component Ratios of Synergistic Combinations Containing Houjari Oleoresin Extracts

**Figure 6:**
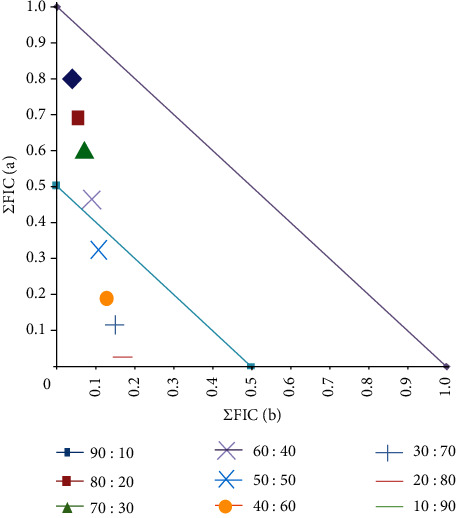
Isobologram for combinations of Houjari (extraction method 2) methanolic extract in with tetracycline against *A. baylyi*. The extract and antibiotic were tested at various ratios against, and results represent mean FIC values of four replicates (*n* = 4). Ratio = % extract:% antibiotic. Ratios lying on or underneath the 0.5 : 0.5 line are considered synergistic (ΣFIC ≤ 0.5). Any points between the 0.5 : 0.5 and 1.0 : 1.0 lines are deemed additive (ΣFIC > 0.5–1.0).

A single combination containing Houjari (extraction method 2) methanolic extract and tetracycline produced synergy and was therefore examined further by isobologram analysis (Figure 6). The results in this isobologram aligned closely with the *y* (conventional antibiotic) axis, indicating that tetracycline was the major contributor to the antibiotic effect of the combination, whereas the extract potentiated its effect. All combinations containing ≤50% of the methanolic Houjari extract component (with ≥50% tetracycline) were synergistic against *A. baylyi.* Thus, these ratios would be most beneficial for treating *A. baylyi* infections.

### 3.5. Quantification of Toxicity

All extracts were initially screened in the *Artemia* nauplii bioassay across a range of concentrations, and LC_50_ values were determined (Table 5). The reference toxin potassium dichromate (1000 *μ*g/mL) was also tested concurrently as a positive control. The potassium dichromate toxin control was rapid in its onset of mortality, inducing nauplii death within the first 3 hours of exposure and 100% mortality was evident following 4–5 hours (results not shown). The *B. sacra* oleoresin extracts were much slower at inducing toxicity, with mortality not substantially different to the seawater negative control at 5 hours. All extracts induced mortality following 24 hours of exposure, albeit only at relatively high concentrations. As plant extracts with LC_50_ values > 1000 *μ*g/mL have previously been defined as nontoxic in this assay [22], all the *B. sacra* oleoresin extracts were deemed to be nontoxic. This was supported by the HDF cell viability assays, which showed that none of the extracts decreased cell viability by ≥50% at 300 *μ*g/mL, thereby confirming that all extracts were nontoxic.

To further evaluate the suitability of the frankincense oleoresin extracts as therapeutic agents, their therapeutic indexes (TIs) were also calculated. We were unable to calculate TIs for the extracts that were inactive against some bacterial species. However, the *B. sacra* oleoresin extracts generally displayed relatively high TIs (>4) when tested against the other bacterial species. Indeed, a TI value of 58.14 was calculated for the grade 2 Najdi methanolic (extraction method 2) extract against *S. newport*. High TI values were also recorded for several extracts against other bacterial pathogens. Thus, there is a relatively large therapeutic window for these extracts against most bacteria tested, indicating that the extracts are safe and promising drug leads.

### 3.6. Nontargeted GC-MS Headspace Analysis of the Frankincense Extracts

The phytochemical profiles of all of the frankincense oleoresin extracts were evaluated and compared using GC-MS headspace analysis. This method of profiling was selected as previous studies have reported an abundance and variety of mono- and sesquiterpenoids in similar extracts produced from the same *B. sacra* cultivars as examined in our study [7], as well as against related *Boswellia* spp. [11, 12]. As many volatile terpenoids have good antibacterial activity [31], they were deemed to be good targets for phytochemical profiling analysis. Optimised GC-MS headspace parameters were developed and used to examine the phytochemical composition of these extracts. Twenty-eight volatile low-molecular mass compounds were identified in one or more of the extracts (Table 6). A comparison of the calculated and measured molecular masses for each compound is also presented in Supplementary Table 1. Notably, all of the extracts had high abundances of *α*-pinene, with substantially higher relative abundances in the methanolic extracts (generally 52–69% of the total volatile compounds detected), compared with the aqueous extracts (29–45% of the total volatile compounds detected). These values are comparable to other recent studies, which have also reported high abundances of *α*-pinene in *B. sacra* [7], as well as in other *Boswellia* spp. [11, 12]. These high levels of *α*-pinene are especially noteworthy as good antibacterial activity has been reported for this terpenoid [31]. Several other volatile terpenoids were also detected in abundance in all oleoresin extracts. p-Cymene, limonene, and *β*-elemene were also relatively abundant in all methanolic and aqueous extracts. Additionally, *α*-terpineol was also detected in most extracts and was in relative abundance in the Sahli and Houjari extracts, but in lower relative abundances in the corresponding Najdi extracts. As all of these terpenoids also have good antibacterial activity [31], it is likely that they may all contribute to the antibacterial activity reported in our study. However, the GC-MS method used in our study is nonquantitative and the % relative abundance values reported herein are a measure of the area under the peak expressed as the % of the total area under all chromatographic peaks. These values do not take into account the different signals from molecular impacts of individual molecules (and thus the variable areas under individual peaks). As such, the % relative abundance values we report in our study are approximate % composition values only, and future studies utilising GC-FID are required to provide a more quantitative evaluation of the levels of the individual components.

## 4. Discussion

This study investigated the ability of *B. sacra* oleoresin extracts to inhibit the growth of some gastrointestinal bacterial pathogens, as well as selected bacterial triggers of autoimmune inflammatory diseases. Notably, several of the *B. sacra* oleoresin extracts were identified as effective growth inhibitors against several bacteria, with the greatest activity associated with the methanolic Najdi oleoresin. Interestingly, the potency of these extracts was substantially greater than that reported for essential oils prepared using similar oleoresins in a recent study [7]. Thus, the *B. sacra* oleoresin extracts examined in our study have potential in the treatment of gastrointestinal infections, as well as in preventing and treating some autoimmune inflammatory diseases (particularly rheumatoid arthritis and ankylosing spondylitis). Interestingly, all of the bacterial species screened in our study were MDR strains. Therefore, the *B. sacra* extracts studied herein may have particular relevance against resistant bacterial infections.

The growth inhibitory activity of the *B. sacra* oleoresin extracts against MDR bacterial strains highlights a couple of interesting possibilities. The extracts may function by different mechanisms to the conventional antibiotics to which these bacteria have developed resistance and may provide a novel therapy. This is an exciting prospect as it would not only allow medical science to treat infections not otherwise treatable by allopathic medicine, but it may also provide new scaffolds for chemical modifications, allowing scientists to enhance the efficacy and bioavailability of the therapies. Alternatively, the extracts may contain potentiating components that allow antibiotic compounds to function at high efficacy, even when the bacterial pathogen is otherwise resistant to that compound. This may be a more promising prospect, as the potentiating component(s) may block or bypass bacterial resistance mechanisms, allowing several existing antibiotics to function again at high efficacy, even in resistant bacterial strains. Indeed, our results indicate that this may indeed be the case for the *B. sacra* oleoresin extracts. Our results demonstrated that the extracts potentiated the activity of several antibiotics and were particularly effective at synergising tetracycline activity. Synergy was also noted in some bacteria when used in combination with chloramphenicol or ciprofloxacin.

Whilst bacteria use numerous resistance mechanisms to decrease the effects of antibiotics, the multidrug-resistant (MDR) efflux pumps is the most commonly used method. Efflux pumps rapidly pump intracellular antibiotics from the cell, thereby reducing the antibiotic concentration, rendering the cell resistant to the effects of the antibiotic [32, 33]. Some efflux pumps (including tetracycline efflux pumps) [2, 30] are specific to a single class of antibiotics and allow the bacterium to avoid the effects of that class of antibiotic only. In contrast, other efflux pumps may allow the bacteria to avoid the effects of several types of antibiotics. Interestingly, many MDR pump inhibitors have been reported in other plant extracts and these allow the plants to enhance the activity of their own antimicrobial compounds. This may explain the lower activity of components isolated from some plant extracts compared with the activity of the crude extract [29]. Such MDR efflux pump inhibitors have potential to treat resistant bacterial infections when used in combination with conventional antibiotics [33]. Indeed, several plant-derived potentiating compounds have already been reported. *Lupinus argenteus* Pursh isoflavone compounds substantially potentiate the activity of the plant-derived antibiotic compound berberine, as well that of the synthetic fluoroquinoline antibiotic norfloxacin against *S. aureus* growth [33]. That study determined that the isoflavones increase the intracellular concentration of berberine in the bacterium by inhibiting its MDR efflux pump. *Mezoneuron benthamianum* (Roxb.) Benth. and *Securinega virosa* Leandri extracts also contain efflux pump inhibitors that block fluoroquinolone, tetracycline, and erythromycin efflux pumps in *S. aureus* strains resistant to methicillin (MRSA) and several other antibiotics, thereby reducing the MIC of the conventional antibiotics by a factor of more than four [32].

Similarly, *Punica granatum* L. methanolic extracts synergise the activity of ampicillin, chloramphenicol, gentamicin, oxacillin, and tetracycline in MRSA and MSSA by inhibiting the NorA MDR efflux pump, thereby increasing the intracellular antibiotic concentration in the cell [34]. By increasing the intracellular antibiotic concentration, the *P. granatum* extracts substantially increase the effects of those antibiotics, extending their useful lifetime [35]. Similarly, the flavone baicalein, which is extracted from *Scutellaria baicalensis* Georgi leaves, also inhibits the NorA efflux pump and potentiates the bactericidal activity of gentamicin [36]. Surprisingly, there are no EPI/antimicrobial drug combinations currently in clinical use, although these combinations may have a profound impact on the efficacy of antibiotic chemotherapies and substantial recent research into identifying potential EPIs has already been published [19, 28, 37–39].

In addition to the compounds already described, many other constituents have been identified as potential efflux pump inhibitors, including several terpenoids, as well as piperine and linoleic acid [39]. Notably, the diterpenoid carnosic acid, which was isolated from *Rosmarinus officinalis* L., strongly potentiates the growth inhibitory activity of erythromycin by inhibiting erythromycin efflux pumps [40]. GC-MS headspace analysis of the methanolic and aqueous *B. sacra* oleoresin extracts identified a diversity and relative abundance of terpenoids. Monoterpenoids were particularly prevalent. Indeed, *α*-pinene accounted for 45–69% of the relative abundance of the volatile compounds identified in all of the *B. sacra* extracts. A diversity of other monoterpenoids were also detected in abundance across all extracts. Monoterpenes have a wide variety of biological effects, including inhibiting the growth of bacterial and fungal pathogens [31]. It is therefore likely that the monoterpenoids in the extracts contribute to the growth inhibitory activity against the bacterial pathogens tested herein. Indeed, many of the monoterpenoids putatively identified in our study have been previously reported to have potent broad-spectrum antibacterial activity [31]. A wide variety of monoterpenoids, including *α*-pinene, terpineol, sabinol, carvone, carveol, borneol, limonene, linalool, thymol, as well as their derivatives, inhibit the growth of an extensive panel of pathogenic bacteria [31]. Further phytochemical evaluation studies and bioactivity-driven isolation of active components are required to evaluate the mechanism(s) of bacterial growth inhibition.

## 5. Conclusions

The *B. sacra* oleoresin extracts evaluated in this study had noteworthy bacterial growth inhibitory activity alone and may therefore be useful in treating gastrointestinal infections and for inhibiting the growth of some bacterial triggers of autoimmune diseases. The antibiotic properties of combinations of the *B. sacra* extracts and conventional antibiotics were substantially higher than that of the individual components alone, even in antibiotic-resistant bacteria. Although the mechanisms of synergy are unclear, compounds within the *B. sacra* extracts (particularly mono- and sesquiterpenoids) may inhibit bacterial efflux pumps, thereby increasing the intracellular concentration of the antibiotic, although this is yet to be verified. The use of combinations of *B. sacra* oleoresin extracts and conventional antibiotics may therefore increase the effectiveness of the antibiotic components and reduce the side effects caused by using high doses of antibiotics, and the use of lower levels of antibiotics may subsequently lessen the development of further drug-resistant pathogens.

## Figures and Tables

**Table 1:** The grades and regions of collection for the frankincense oleoresin cultivars examined in this study

Sample code	Region	Species	Collection site	Cultivar	Oleoresin grade	Voucher code
1	Dhofar plateau region	*B. sacra*	Wadi Doka (42km south of Salalah centre)	Najdi	1	GU-BS12a1-17
2					2	GU-BS12b2-17
3					3	GU-BS12c3-17

4	Dhofar valleys region	*B. sacra*	Al Magseel (47 km west of Salalah centre)	Sahli (Shaebi)	1	GU-BS13a1-17
5					2	GU-BS13b2-17
6					3	GU-BS13c3-17

7	Samhan mountains	*B. sacra*	Jabal Samah (86.9 km from Salalah centre)	Houjari	1	GU-BS14a1-17
8					2	GU-BS14b2-17
9					3	GU-BS14b3-17

**Table 2:** The mass of dried extracted material, the concentration after resuspension in deionised water (containing 1% DMSO), and qualitative phytochemical screenings frankincense oleoresin extracts.

Sample code	Extract solvent	Mass of dried extract (mg)	Concentration of resuspended extract (mg/mL)	Total phenolics	Water-soluble phenolics	Water-insoluble phenolics	Cardiac glucosides	Saponins	Triterpenoids	Phytosterols	Alkaloids	Flavonoids	Tannins	Free anthraquinones	Combined anthraquinones
1	*M*	687	68.7	++	++	+	−	++	+	+	−	+++	−	−	−
	*W*	338	33.8	++	++	+	−	++	++	+	−	++	−	−	−
2	*M*	596	59.6	++	++	−	−	++	++	+	−	++	−	−	−
	*W*	385	38.5	++	++	+	−	++	+	+	−	++	−	−	−
3	*M*	623	62.3	++	+	+	−	+	+	+	−	++	−	−	−
	*W*	404	40.4	++	++	−	−	++	+	+	−	++	−	−	−
4	*M*	593	59.3	++	++	+	−	++	++	+	−	++	−	−	−
	*W*	348	34.8	++	++	+	−	++	+	+	−	++	−	−	−
5	*M*	573	57.3	++	++	−	−	++	++	+	−	++	−	−	−
	*W*	322	32.2	++	++	+	−	++	+	+	−	++	−	−	−
6	*M*	545	54.5	++	++	−	−	+	+	+	−	++	−	−	−
	*W*	394	39.4	++	+	+	−	++	+	+	−	++	−	−	−
7	*M*	640	64	++	++	+	−	++	++	+	−	++	−	−	−
	*W*	276	27.6	++	++	+	−	++	++	+	−	+++	−	−	−
8	*M*	587	58.7	++	++	+	−	++	+	+	−	++	−	−	−
	*W*	306	30.6	++	++	+	−	++	++	+	−	++	−	−	−
9	*M*	570	57	++	+	−	−	+	+	+	−	++	−	−	−
	*W*	345	34.5	++	++	+	−	++	+	+	−	++	−	−	−

*Notes.* +++ indicates a large response; ++ indicates a moderate response; + indicates a minor response; − indicates no response in the assay. *M* = methanolic extract; *W* = aqueous extract.

**Table 3:** MIC values (*μ*g/mL) for the *B. sacra* oleoresin extracts against selected bacterial pathogens.

				Gastrointestinal pathogens					Autoimmune inflammatory trigger bacteria		
				*E. coli*	*E. faecalis*	*S. aureus*	*S. newport*	*S. sonnei*	*P. mirabilis*	*K. pneumoniae*	*A. baylyi*	*P. aeruginosa*
Najdi	Method 1	*M*	DD	2483	**850**	1128	**626**	1540	**675**	**783**	1086	2186
			LD	1165	**476**	**628**	**146**	1348	**605**	**625**	**805**	2250
		*W*	DD	—	—	2550	3263	—	1284	2568	—	—
			LD	2750	1860	1876	1245	—	1550	2250	1875	—
	Method 2	*M*	DD	1170	**476**	**521**	**147**	1256	**477**	**228**	**736**	1899
			LD	**826**	**225**	**326**	**86**	**920**	**280**	**126**	**437**	1558
		*W*	DD	—	—	2860	1280	—	**583**	**576**	1873	—
			LD	1890	1387	1256	**863**	2540	**625**	**528**	**920**	—
	Method 3	*M*	DD	**896**	**625**	**625**	**526**	1386	**487**	**428**	**588**	1860
			LD	**526**	**376**	**326**	**128**	**664**	**225**	**258**	**376**	1280
		*W*	DD	—	2568	2803	2560	—	**970**	**826**	1080	—
			LD	1566	—	1440	1280	—	**486**	**120**	**500**	—
Sahli	Method 1	*M*	DD	2287	**927**	1883	**825**	2250	1827	1250	>5000	—
			LD	1680	**475**	**826**	**368**	1858	1250	1125	2500	2285
		*W*	DD	—	—	—	—	—	—	—	—	—
			LD	—	1854	2256	1440	—	—	—	—	—
	Method e 2	*M*	DD	1568	**876**	1059	**437**	1405	**835**	**825**	2583	—
			LD	1125	**283**	**528**	**128**	**876**	**750**	**576**	1265	2650
		*W*	DD	—	—	—	2500	—	—	2836	—	—
			LD	—	—	—	1868	—	—	1560	>5000	—
	Method 3	*M*	DD	**920**	**827**	1298	**788**	2568	**625**	**528**	1287	—
			LD	**765**	**358**	**625**	**246**	1280	**574**	**325**	**870**	1560
		*W*	DD	—	—	—	3285	—	—	2256	2862	—
			LD	—	—	—	2500	—	>5000	1280	1255	—
Houjari	Method 1	*M*	DD	1830	**450**	1276	1540	1863	**927**	1045	1653	—
			LD	**854**	**128**	**665**	1194	1280	**825**	**805**	1295	1875
		*W*	DD	1280	**856**	—	—	—	1580	1293	—	—
			LD	**874**	**680**	1128	2580	3860	1135	**960**	>5000	—
	Method 2	*M*	DD	1147	**540**	**920**	**883**	1086	**625**	**459**	**685**	2279
			LD	**486**	**68**	**376**	**750**	**826**	**480**	**275**	**775**	**876**
		*W*	DD	2280	1580	—	—	—	—	2876	1287	—
			LD	1488	**726**	>5000	1478	1886	2586	2280	1476	—
	Method 3	*M*	DD	**827**	**525**	1352	1855	1455	1250	**400**	1180	—
			LD	**628**	**156**	**728**	1480	**926**	**750**	**325**	**838**	1358
		*W*	DD	—	1156	—	—	—	—	2500	—	—
			LD	—	**800**	>5000	>5000	>5000	—	1186	1586	—

LD antibiotic controls MIC values		Penicillin	2.5	**0.63**	—	1.25	1.25	1.25	1.25	2.5	—
		Erythromycin		1.25	**0.63**	2.5	1.25	2.5	1.25	2.5	**0.63**	—
		Chloramphenicol	1.25	1.25	1.25	1.25	**0.64**	**0.63**	1.25	**0.16**	2.5
		Ciprofloxacin	**0.32**	**0.08**	**0.64**	**0.64**	**0.64**	**0.08**	**0.08**	**0.08**	**0.32**
		Tetracycline	1.25	1.25	1.25	2.5	1.25	1.25	**0.32**	**0.32**	—

*M* = methanolic extract; *W* = aqueous extract; DD = disc diffusion MIC value; LD = liquid dilution MIC value; Methods 1, 2, and 3 refer to the extraction method used; — indicates that no inhibitory activity was detected at any concentration tested. Extracts with noteworthy activity (MIC < 1000 *μ*g/mL) or antibiotics (MIC <1 *μ*g/mL) are indicated in bold. Bacteria are considered susceptible to conventional antibiotics if MIC ≤ 1 *μ*g/mL.

**Table 4:** Combinational effects of the *B. sacra* oleoresin extracts and conventional antibiotics measured as the sum of the fractional inhibition concentrations (ΣFIC).

Extract component			Antibiotic component	Gastrointestinal pathogens					Autoimmune inflammatory trigger bacteria			
				*E. coli*	*E. faecalis*	*S. aureus*	*S. newport*	*S. sonnei*	*P. mirabilis*	*K. pneumoniae*	*A. baylyi*	*P. aeruginosa*
Najdi	Method 1	*M*	Pen	—	3.71	—	1.64	3.62	1.93	1.75	—	—
			Erth	4.5	*1*	—	—	*1*	*1*	—	1.3	—
			Chl	*1*	4.5	*1*	**0.5**	*1*	1.56	1.12	2.4	1.12
			Cip	*0.76*	2.02	**0.5**	*0.51*	*0.99*	3	2.63	1.03	*1*
			Tet	*0.73*	14.7	5	4.06	*0.53*	1	**0.38**	**0.47**	—
		*W*	Pen	—	1.69	—	2.11	—	2.2	2.27	2.65	—
			Erth	—	1.85	—	—	—	1.49	—	1.87	—
			Chl	.	1.29	1.82	1.34	—	1.53	1.37	1.52	—
			Cip	—	1.04	*0.77*	*0.97*	—	2.28	1.93	*0.96*	—
			Tet	—	3.72	2.26	2.46	—	1.06	1.46	*0.75*	—
	Method 2	*M*	Pen	—	2.68	—	3.27	2.44	3.15	2.18	2.1	—
			Erth	5.1	1.27	—	—	1.36	1.15	—	1.73	—
			Chl	*0.86*	4.13	1.09	**0.46**	*0.91*	1.81	1.36	1.88	1.47
			Cip	*0.68*	1.72	**0.44**	*0.58*	*0.58*	1.96	1.95	1.09	*1*
			Tet	1.47	5.63	6.36	3.74	1.02	*0.96*	**0.47**	**0.35**	—
		*W*	Pen	—	2.25	—	2.78	2.88	2.87	—	1.96	—
			Erth	3.11	1.83	—	—	1.7	1.29	—	1.58	—
			Chl	2.76	1.14	2.14	1.36	2.25	1.41	—	1.37	—
			Cip	1.04	1.07	1.26	*0.92*	*0.86*	1.03	—	1.05	—
			Tet	1.74	2.73	2.49	1.49	2.37	1.82	—	*0.89*	—
	Method 3	*M*	Pen	—	2.11	—	1.88	1.83	2.7	2.81	2.83	—
			Erth	4.1	1.83	—	—	1.28	1.2	—	1.55	—
			Chl	1.36	5.01	1.47	**0.5**	*0.67*	1.34	1.59	2.06	1.63
			Cip	*0.92*	1.45	**0.48**	*0.65*	*0.52*	1.45	1.38	1.36	*0.95*
			Tet	*0.86*	4.79	4.16	2.66	1.46	*0.83*	**0.5**	**0.5**	—
		*W*	Pen	—	3.08	—	2.92	—	2.25	3.17	3.4	—
			Eryth	2.92	1.54	—	—	—	1.86	—	1.72	—
			Chl	1.46	2.09	1.37	2.4	—	1.17	1.84	2.62	—
			Cip	1.17	1.46	1.66	1.22	—	1.19	1.5	1.19	—
			Tet	3.31	3.02	2.17	2.86	—	1.59	1.29	1.05	—
Sahli	Method 1	*M*	Pen	—	3.26	—	1.87	—	2.48	2.2	1.72	—
			Erth	—	*1*	—	—	—	*1*	—	*0.81*	—
			Chl	—	3.5	*1*	1.12	—	*0.65*	1.12	2.5	—
			Cip	—	1.01	*0.51*	*0.88*	—	2.02	5.26	1.03	—
			Tet	—	**0.21**	1.06	**0.43**	—	*1*	*0.71*	**0.19**	—
		*W*	Pen	—	2.81	—	2.13	—	—	—	—	—
			Erth	—	1.94	—	—	—	—	—	—	—
			Chl	—	1.62	—	1.88	—	—	—	—	—
			Cip	—	*0.93*	—	1.47	—	—	—	—	—
			Tet	—	2.77	—	2.16	—	—	—	—	—
	Method 2	*M*	Pen	—	2.09	—	2.5	1.74	3.17	2.82	1.68	—
			Erth	4.5	1.16	—	—	1.16	1.05	—	*0.92*	—
			Chl	*1*	2.67	1.21	*1*	1.31	*0.85*	1.62	1.92	2.81
			Cip	1.52	1.28	*0.73*	*0.63*	*0.85*	1.74	4.31	1.37	1
			Tet	*0.81*	**0.42**	1.47	**0.25**	*0.88*	*0.96*	*0.92*	**0.36**	—
		*W*	Pen	—	—	—	1.66	—	2.8	2.13	3.7	—
			Erth	—	—	—	—	—	2.16	—	2.87	—
			Chl	—	—	—	1.24	—	1.49	1.07	1.4	—
			Cip	—	—	—	1.27	—	1.27	1.73	1.06	—
			Tet	—	—	—	*0.78*	—	1.08	1.38	1.35	—
	Method 3	*M*	Pen	—	3.06	—	1.88	1.31	2.48	2.68	3.15	—
			Erth	5.14	1.18	—	—	1.96	1.32	—	*0.75*	—
			Chl	*0.97*	2.06	1.36	1.63	2.87	*0.77*	2.14	1.38	2.4
			Cip	1.14	*0.98*	*0.74*	*0.92*	*0.73*	1.59	4.16	*0.92*	*1*
			Tet	1.16	**0.41**	1.19	**0.44**	1.16	*1*	*0.92*	**0.47**	—
		*W*	Pen	—	—	—	1.46	—	—	2.82	3.35	—
			Erth	—	—	—	—	—	—	—	1.54	—
			Chl	—	—	—	2.79	—	—	1.59	1.36	—
			Cip	—	—	—	1.62	—	—	1.69	*0.98*	—
			Tet	—	—	—	1.23	—	—	1.48	1.06	—
Houjari	Method 1	*M*	Pen	—	2.31	—	3.65	2.48	1.95	1.86	1.36	—
			Erth	4.5	*1*	—	—	1.13	1	—	*0.54*	—
			Chl	*1*	3.51	1.06	*1*	1.13	2.5	1.12	1.6	2.81
			Cip	2.03	2.02	*0.51*	*0.51*	*0.83*	1.87	2.94	*0.59*	*1*
			Tet	*0.55*	*0.65*	1.06	3.56	*0.75*	*1*	*0.51*	**0.49**	—
		*W*	Pen	—	3.62	—	2.6	1.82	3.16	2.51	—	—
			Erth	2.97	2.18	—	—	2.43	1.65	—	—	—
			Chl	1.46	1.23	1.47	1.82	1.59	1.18	1.77	—	—
			Cip	*0.88*	1.17	1.06	1.44	1.64	*0.98*	1.25	—	—
			Tet	1.87	2.14	2.18	1.52	2.83	1.13	1.47	—	—
	Method 2	*M*	Pen	—	3.62	—	2.78	3.16	2.41	2.2	2.02	—
			Erth	4.21	*0.98*	—	—	1.48	*0.92*	—	*0.75*	—
			Chl	*0.76*	2.26	*0.98*	1.25	1.72	1.87	1.35	1.83	2.2
			Cip	1.26	1.53	*0.62*	*0.86*	*0.73*	1.19	2.16	*0.74*	*0.95*
			Tet	0.83	*0.92*	1.83	2.43	1.07	*1*	*0.75*	**0.38**	—
		*W*	Pen	—	2.83	—	1.89	2.77	—	—	3.16	—
			Erth	2.46	1.95	—	—	1.42	—	—	1.85	—
			Chl	1.25	1.07	—	1.65	1.86	—	—	1.49	—
			Cip	*0.96*	*1*	—	1.1	2.19	—	—	1.11	—
			Tet	1.48	1.89	—	3.16	3.4	—	—	1.32	—
	Method 3	*M*	Pen	—	3.16	—	1.47	1.96	1.78	2.92	1.83	—
			Erth	5.67	1.22	—	—	2.01	*0.96*	—	1.06	—
			Chl	*0.93*	1.83	*0.94*	2.62	1.83	2.43	1.16	1.48	2.54
			Cip	1.05	1.29	*0.57*	*0.98*	*0.79*	1.76	*0.97*	*0.86*	1
			Tet	0.59	*0.85*	1.38	1.74	1.24	*0.95*	*0.78*	**0.48**	—
		*W*	Erth	—	3.2	—	—	—	2.97	2.25	2.89	—
			Erth	—	2.07	—	—	—	1.48	—	1.84	—
			Chl	—	2.49	—	—	—	1.92	1.08	1.11	—
			Cip	—	1.88	—	—	—	*1*	1.07	*0.94*	—
			Tet	—	1.45	—	—	—	1.46	1.83	1.27	—

Synergistic ∑FIC values (0–≤0.5) are shown in bold; additive values (>0.5-≤1.0) are shown in italics; indifferent values (>1.0–≤4.0) are shown in normal text; antagonistic interactions (>4.0) are shown in underlined. Method refers to the extraction method used; M = methanolic extract; *W* = aqueous extract; areas with “—”indicate combinations that were not tested as the antibiotic MIC ≥ 2.5 *μ*g/mL or if extract MIC was ≥2000 *μ*g/mL. Pen = penicillin; Erth = erythromycin; Chl = chloramphenicol; Cip = ciprofloxacin; Tet = tetracycline.

**Table 5:** Toxicity evaluation (expressed as LC_50_ in *μ*g/mL) by ALA and HDF cytotoxicity assay and calculation of therapeutic index.

			Toxicity		Therapeutic index (TI)								
			ALA LC_50_ (*μ*g/mL)	HDF cytotoxicity	*E. coli*	*E. faecalis*	*S. aureus*	*S. newport*	*S. sonnei*	*P. mirabilis*	*K. pneumoniae*	*A. baylyi*	*P. aeruginosa*
Najdi	Method 1	*M*	>5000	NT	4.29	**10.5**	7.96	**34.25**	3.71	8.26	8	6.21	2.22
		*W*	4483	NT	3.85	2.41	2.39	3.6	CND	2.89	1.99	2.39	CND
	Method 2	*M*	>5000	NT	6.05	**22.22**	**15.34**	**58.14**	4.87	**17.86**	**39.68**	**11.44**	3.21
		*W*	4792	NT	2.54	3.45	3.82	5.55	1.89	7.67	9.08	5.21	CND
	Method 3	*M*	>5000	NT	9.51	**13.3**	**15.34**	**39.06**	7.53	**17.11**	**19.38**	**13.3**	3.91
		*W*	4186	NT	2.67	CND	2.91	3.27	CND	8.61	**34.88**	8.37	CND
Sahli	Method 1	*M*	>5000	NT	2.98	**10.53**	6.05	**13.59**	2.69	4	4.44	2	2.19
		*W*	>5000	NT	CND	2.7	2.22	3.47	CND	CND	CND	CND	CND
	Method 2	*M*	>5000	NT	4.44	**17.67**	9.47	**39.06**	5.71	6.67	8.68	3.95	CND
		*W*	4787	NT	CND	CND	CND	2.56	CND	CND	3.07	≤1	CND
	Method 3	*M*	>5000	NT	6.54	14	8	**20.33**	3.91	8.71	15.38	5.75	3.21
		*W*	4460	NT	CND	CND	CND	1.78	CND	0.89	3.48	3.55	CND
Houjari	Method 1	*M*	>5000	NT	5.85	**39.06**	**13.3**	4.19	3.91	6.06	6.21	3.86	2.67
		*W*	>5000	NT	5.72	7.35	4.43	1.94	1.3	4.41	5.21	≤1	CND
	Method 2	*M*	>5000	NT	**10.29**	**73.53**	**13.3**	6.67	6.05	**10.42**	**18.19**	6.45	5.71
		*W*	>5000	NT	3.36	6.89	≤1	3.38	2.65	2.19	2.19	3.39	CND
	Method 3	*M*	>5000	NT	7.96	**32.05**	6.97	3.38	5.4	6.67	**15.38**	5.97	3.68
		*W*	>5000	NT	CND	6.25	≤1	≤1	≤1	CND	4.22	3.15	CND

Controls	Potassium dichromate		83	WNT	WNT								
	Quinine		WNT	31.4									

ALA = *Artemia* lethality assay; HDF = human dermal fibroblasts; Method refers to the extraction method used; *M* = methanolic extract; *W* = aqueous extract; NT = non toxic; CND = could not determine as the extract was inactive at all concentrations tested; WNT = was not tested. Notable TI values (>10) are indicated with bold text. Here, ALA > 5000 *μ*g/mL, 5000 *μ*g/mL is taken as the LC_50_ for TI calculations.

**Table 6:** GC-MS analysis of the *B. sacra* oleoresin extracts, putative identification, and relative abundance (%) of each compound.

Compound	Retention time (min)	Najdi oleoresin						Sahli oleoresin						Houjari oleoresin					
		Method 1		Method 2		Method 3		Method 1		Method 2		Method 3		Method 1		Method 2		Method 3	
		*M*	*W*	*M*	*W*	*M*	*W*	*M*	*W*	*M*	*W*	*M*	*W*	*M*	*W*	*M*	*W*	*M*	*W*
2,4(10)-Thujadiene	12.501	0.08	—	0.27	—	0.22	—	0.14	—	0.47	—	0.59	0.05	0.56	0.02	0.01	—	0.47	—
p-Cymene	15.26	2.55	2.13	2.71	2.47	3.46	2.42	1.12	0.95	1.48	1.27	2.17	1.89	1.87	1.54	2.26	1.98	4.2	3.76
Limonene	15.497	1.38	3.41	1.76	3.86	2.27	5.5	2.55	4.78	3.32	6.2	3.78	8.15	1.13	1.96	2.58	5.68	5.26	12.7
*γ*-Terpineol	15.94	0.1	—	0.28	—	0.37	—	0.46	—	1.12	—	1.67	—	0.72	—	1.21	—	1.18	—
*α*.-Pinene	16.436	68.6	44.6	62.7	41.7	51.7	43.8	66.5	38.3	63.1	38.8	59.2	40.3	54.7	34.2	51.6	31.4	45.7	28.6
p-Cymenene	17.454	0.32	—	0.48	—	0.4	—	0.47	—	0.96	—	0.78	—	0.25	—	0.62	—	0.33	—
Linalool	17.764	0.16	0.3	0.47	0.52	0.28	0.31	1.13	1.26	0.54	0.49	0.48	0.57	1.35	1.48	1.14	1.11	1.14	1.27
Thujone	18.368	0.21	0.37	0.52	0.72	0.83	1.05	0.27	0.38	0.78	0.92	1.16	1.27	0.44	0.57	0.26	0.33	0.81	1.07
Verbenol	18.672	0.13	0.28	0.55	0.83	0.24	0.47	1.21	1.36	0.92	1.11	0.77	0.94	2.16	2.32	1.88	2.08	1.14	1.18
(+)-Sabinol	19.132	0.53	0.76	1.1	1.35	1.31	1.88	1.24	1.43	1.75	2.16	3.69	4.88	0.12	0.17	0.86	1.14	0.16	0.36
p-Cymen-8-ol	19.433	0.06	0.18	0.11	0.23	0.08	0.14	0.09	0.15	0.52	1.03	0.43	0.74	0.46	1.11	1.32	2.28	0.71	1.46
Pinocarvone	19.885	0.21	0.31	0.32	0.18	0.27	0.34	0.17	0.28	0.34	0.26	0.33	0.35	1.3	1.88	2.42	2.62	1.28	1.48
*α*-Terpineol	20.002	0.09	—	1.46	0.83	1.81	1.17	0.27	—	1.38	0.92	1.47	1.05	1.16	0.71	1.95	1.26	1.78	1.03
Verbenone	20.186	0.92	0.73	0.74	0.48	0.44	0.24	0.83	0.9	1.33	1.18	0.92	0.82	2.76	2.48	3.26	2.87	2.27	1.93
Terpinen-4-ol	20.361	1.21	1.37	2.36	1.15	4.22	3.76	1.47	1.68	2.71	3.21	2.57	2.84	3.22	3.41	5.18	5.57	6.3	6.83
p-Cymen-8-ol	20.559	—	—	—	—	0.47	—	—	—	0.57	0.62	0.6	0.55	0.83	0.66	1.84	2.31	1.26	1.42
*α*-Terpineol	20.768	0.08	—	0.27	0.22	0.64	0.49	0.93	1.13	2.26	1.83	3.58	2.36	1.15	0.89	1.74	1.46	2.83	1.73
Sabinol	21.144	—	—	0.86	0.57	0.28	0.16	—	—	0.97	0.72	1.47	0.86	—	—	0.73	0.55	0.27	0.43
*β*-Elemene	21.407	0.12	0.18	3.7	5.2	6.83	8.11	0.75	1.16	4.25	4.75	7.42	8.28	1.87	2.37	4.83	5.72	4.28	5.17
Carveol	21.615	1.08	0.56	1.76	1.2	1.94	1.13	0.52	0.26	0.77	0.31	1.18	0.68	0.35	—	0.47	—	0.64	0.18
Carvacrol	22.392	—	—	—	0	0.21	0.07	0.08	—	—	—	—	—	0.22	—	0.42	0.16	0.26	0.14
(-)-Bornyl acetate	23.672	0.87	—	1.75	0.27	2.2	0.41	0.92	—	1.55	—	2.06	0.51	1.19	0.13	1.42	0.3	2.44	0.63
Thymol	24.022	0.24	0.06	0.47	0.13	0.12	0.06	0.36	0.18	0.45	0.22	0.15	0.1	0.44	0.1	1.12	0.36	0.47	0.18
Caryophyllene	28.572	0.23	—	1.36	0.14	0.37	—	0.76	0.08	1.47	0.12	1.15	0.12	0.77	0.12	1.91	0.15	1.26	0.27
Humulene	30.016	0.11	—	0.85	0.07	1.26	0.11	0.24	—	0.41	0.06	0.86	0.13	0.3	—	0.52	0.08	0.96	0.17
*γ*-Muurolene	30.75	0.04	—	0.06	—	0.04	—	0.03	—	0.07	—	0.07	—	0.15	—	0.22	—	0.25	—
Epicubenol	34.623	—	—	0.26	—	0.89	0.11	—	—	0.13	—	0.24	—	—	—	0.72	0.14	1.26	0.19
*τ*-Cadinol	34.856	0.16	—	0.14	—	0.26	—	0.18	—	0.22	—	0.25	—	0.28	—	0.16	—	0.54	0.13

The relative abundance expressed in this table is a measure of the area under the peak expressed as the % of the total area under all chromatographic peaks. Method refers to the extraction method used.

## Data Availability

All data are presented in this study and are available from the corresponding author upon request.

## Abbreviations

ALA: Brine-shrimp (*Artemia*) lethality assay
DMSO: Dimethyl sulfoxide
GC-MS: Gas chromatography-mass spectrometry
HDF: Normal human dermal fibroblasts
INT: *ρ*-Iodonitrotetrazolium chloride
LD_50_: Dose of sample necessary that causes death of 50% of test organisms or cells
MDR: Multidrug resistant
MIC: Minimum inhibitory concentration
MRSA: Methicillin-resistant *Staphylococcus aureus*
MSSA: Methicillin-sensitive *Staphylococcus aureus*
TDR: Totally drug resistant
WHO: World Health Organisation
ZOI: Zone of inhibition.

## Additional Points

(i) *Boswellia sacra* extracts were prepared from three cultivars (Najdi, Sahli, and Houjari) and screened against a panel of bacterial pathogens.(ii) Methanolic *B. sacra* extracts of all cultivars displayed broad-spectrum antibacterial activity.(iii) The *B. sacra* extracts were also screened in combination with conventional antibiotics, and the ΣFIC and class of interaction were determined. (iV) The synergistic combinations were further evaluated by isobologram analysis, and the synergistic ratios were determined. (v) All *B. sacra* oleoresin extracts were nontoxic in the *Artemia* nauplii and HDF cell viability bioassays. (v) GC-MS headspace analysis identified an abundance of mono- and sesquiterpenoids in the extracts and highlighted *α*-pinene as the major component.

## Supplementary Materials

Supplementary Materials Compounds putatively identified in *B. sacra* oleoresins by GC-MS analysis, their empirical formula, and the comparison of the calculated and measured molecular masses.

## References

[1] World Health Organisation (2016). *Antimicrobial Resistance*.

[2] Cheesman M. J., Ilanko A., Blonk B., Cock I. E. (2017). Developing new antimicrobial therapies: are synergistic combinations of plant extracts/compounds with conventional antibiotics the solution?. *Pharmacognosy Reviews*.

[3] Al-Yasiry A. R. M., Kiczorowska B. (2016). Frankincense-therapeutic properties. *Postępy Higieny I Medycyny Doświadczalnej*.

[4] Iram F., Khan S. A., Husain A. (2017). Phytochemistry and potential therapeutic actions of Boswellic acids: a mini-review. *Asian Pacific Journal of Tropical Biomedicine*.

[5] Verho M., Seitz S., Paul M. (2014). Tetra- and pentacyclic triterpene acids from the ancient anti-inflammatory remedy frankincense as inhibitors of microsomal prostaglandin E2 synthase-1. *Journal of Natural Products*.

[6] Van Vuuren S. F., Kamatou G. P. P., Viljoen A. M. (2010). Volatile composition and antimicrobial activity of twenty commercial frankincense essential oil samples. *South African Journal of Botany*.

[7] Di Stefano V., Schillaci D., Grazia C., Rishan M., Rashan L. (2020). *In vitro* antimicrobial activity of frankincense oils from *Boswellia sacra* grown in different locations of the Dhofar region (Oman). *Antibiotics*.

[8] Ljaljevic Grbic M., Unkovic N., Dimkic I. (2018). Frankincense and myrrh essential oils and burn incense fume against micro-inhabitants of sacral ambients. Wisdom of the ancients?. *Journal of Ethnopharmacology*.

[9] El-Nagerabi S. A. F., Elshafie A. E., AlKhanjari S. S., Al-Bahry S. N., Elamin M. R. (2013). Biological activities of *Boswellia sacra* extracts on the growth and aflatoxins secretion of two aflatoxigenic species of *Aspergillus* species. *Food Control*.

[10] Camarda L., Dayton T., Di Stefano V., Pitonzo R., Schillaci D. (2007). Chemical composition and antimicrobial activity of some oleogum resin essential oils fromBoswellia SPP. (Burseraceae). *Annali di Chimica*.

[11] Biggs I., Sirdaarta J., White A., Edwin Cock I. (2016). GC-MS Analysis of frankincense extracts which inhibit the growth of bacterial triggers of selected autoimmune diseases. *Pharmacognosy Communications*.

[12] Zhang J., Biggs I., Sirdaarta J., White A., Cock I. E. (2016). Antibacterial and anticancer properties of *Boswellia carteri* Birdw. and *Commiphora molmol* Engl. oleo-resin solvent extractions. *Pharmacognosy Communications*.

[13] Kalt F. R., Cock I. E. (2014). Gas chromatography-mass spectroscopy analysis of bioactive *Petalostigma* extracts: toxicity, antibacterial and antiviral activities. *Pharmacognosy Magazine*.

[14] Sautron C., Cock I. E. (2014). Antimicrobial activity and toxicity of *Syzygium australe* and *Syzygium leuhmannii* fruit extracts. *Pharmacognosy Communication*.

[15] Vesoul J., Cock I. (2012). The potential of bunya nut extracts as antibacterial functional food agents. *Pharmacognosy Communications*.

[16] Clinical and Laboratory Standards Institute (2019). *Performance Standards for Antimicrobial Susceptibility Testing*.

[17] Hart C., Ilanko P., Sirdaarta J. (2014). *Tasmannia stipitata* as a functional food/natural preservative: antimicrobial activity and toxicity. *Pharmacognosy Communications*.

[18] Hübsch Z., Van Zyl R. L., Cock I. E., Van Vuuren S. F. (2014). Interactive antimicrobial and toxicity profiles of conventional antimicrobials with Southern African medicinal plants. *South African Journal of Botany*.

[19] Ilanko A., Cock I. E. (2019). The interactive antimicrobial activity of conventional antibiotics and *Petalostigma* spp. extracts against bacterial triggers of some autoimmune inflammatory diseases. *Pharmacognosy Journal*.

[20] Hutchings A., Cock I. E. (2018). An interactive antimicrobial activity of *Embelica officinalis* Gaertn. fruit extracts and conventional antibiotics against some bacterial triggers of autoimmune inflammatory diseases. *Pharmacognosy Journal*.

[21] Ruebhart D. R., Wickramasinghe W., Cock I. E. (2009). Protective efficacy of the antioxidants vitamin E and trolox AgainstMicrocystis aeruginosaand microcystin-LR inArtemia franciscanaNauplii. *Journal of Toxicology and Environmental Health, Part A*.

[22] Cock I. E., Ruebhart D. R. (2009). Comparison of the brine shrimp nauplii bioassay and the ToxScreen-II test for the detection of toxicity associated with *Aloe vera* (*Aloe barbadensis* Miller) leaf extract. *Pharmacognosy Research*.

[23] Shalom J., Cock I. E. (2018). Terminalia ferdinandianaExell. Fruit and leaf extracts inhibit proliferation and induce apoptosis in selected human cancer cell lines. *Nutrition and Cancer*.

[24] Wright M. H., Sirdaarta J., White A., Greener A. C., Cock I. E. (2017). GC-MS headspace analysis of *Terminalia ferdinandiana* fruit and leaf extracts which inhibit *Bacillus anthracis* growth. *Pharmacognosy Journal*.

[25] Cock IE I. E., Cheesman M. (2019). The potential of plants of the genus *Syzygium* (Myrtaceae) for the prevention and treatment of arthritic and autoimmune diseases. *Bioactive Food as Dietary Interventions for Arthritis and Related Inflammatory Diseases*.

[26] Mandeville A., Cock I. E. (2018). *Terminalia chebula* Retz. fruit extracts inhibit bacterial triggers of some autoimmune diseases and potentiate the activity of tetracycline. *Indian Journal of Microbiology*.

[27] Lai W., Chen J., Cock I. E., Cheesman M. J. (2018). The interactive antimicrobial activity of *Withania somnifera* (L.) Dunal root extracts and conventional antibiotics against some bacterial triggers of autoimmune inflammatory diseases. *Pharmacognosy Communications*.

[28] Ilanko P., McDonnell P. A., Van Vuuren S., Cock I. E. (2019). Interactive antibacterial profile of *Moringa oleifera* Lam. extracts and conventional antibiotics against bacterial triggers of some autoimmune inflammatory diseases. *South African Journal of Botany*.

[29] Cock I. E. (2018). Is the pharmaceutical industry’s preoccupation with the monotherapy drug model stifling development of effective new drug therapies?. *Inflammopharmacology*.

[30] Kim T. H., Raiz A., Unni A. D. (2020). Combating antibiotic‐resistant gram‐negative bacteria strains with tetracycline‐conjugated carbon nanoparticles. *Advanced Biosystems*.

[31] Cock I. E. (2013). The phytochemistry and chemotherapeutic potential of *Tasmannia lanceolata* (Tasmanian pepper): a review. *Pharmacognosy Communications*.

[32] Dickson R. A., Houghton P. J., Hylands P. J., Gibbons S. (2006). Antimicrobial, resistance-modifying effects, antioxidant and free radical scavenging activities ofMezoneuron benthamianum Baill.,Securinega virosa Roxb. &Wlld. andMicroglossa pyrifolia Lam. *Phytotherapy Research*.

[33] Morel C., Stermitz F. R., Tegos G., Lewis K. (2003). Isoflavones as potentiators of antibacterial activity. *Journal of Agricultural and Food Chemistry*.

[34] Braga L. C., Leite A. A. M., Xavier K. G. S. (2005). Synergic interaction between pomegranate extract and antibiotics against *Staphylococcus aureus*. *Canadian Journal of Microbiology*.

[35] Pal M. J., Saha BP B. P. (2005). Antimicrobial action of the leaf extract of *Moringa oleifera* Lam. *Ancient Science of Life*.

[36] Chan B. C. L., Ip M., Lau C. B. S. (2011). Synergistic effects of baicalein with ciprofloxacin against NorA over-expressed methicillin-resistant *Staphylococcus aureus* (MRSA) and inhibition of MRSA pyruvate kinase. *Journal of Ethnopharmacology*.

[37] Nel A. L., Murhekar S., Matthews B., White A., Cock I. E. (2020). The interactive antimicrobial activity of Terminalia sericea Burch ex DC. leaf extracts and conventional antibiotics against bacterial triggers of selected autoimmune inflammatory diseases. *South African Journal of Botany*.

[38] Cheesman M. J., White A., Matthews B., Cock I. E. (2019). Terminalia ferdinandiana fruit and leaf extracts inhibit methicillin-resistant *Staphylococcus aureus* growth. *Planta Medica*.

[39] Mikulášová M., Chovanová R., Vaverková Š. (2016). Synergism between antibiotics and plant extracts or essential oils with efflux pump inhibitory activity in coping with multidrug-resistant Staphylococci. *Phytochemistry Reviews*.

[40] Abreu A. C., McBain A. J., Simoes M. (2015). Plants as sources of new antimicrobials and resistance-modifying agents. *Natural Product Reports*.

