# Supplementary material for: Chemical Composition, Antibacterial Activity, and Antibiotic Potentiation of Boswellia sacra Flueck. Oleoresin Extracts from the Dhofar Region of Oman
Source: Evid Based Complement Alternat Med. 2021 May 24;2021:9918935. doi: 10.1155/2021/9918935 (PMC8169251; doi:10.1155/2021/9918935)
Supplement: Supplementary Materials — Compounds putatively identified in B. sacra oleoresins by GC-MS analysis, their empirical formula, and the comparison of the calculated and measured molecular masses. [file 9918935.f1.docx]

**Supplementary Information for:**

**Chemical composition, antibacterial activity and antibiotic potentiation of *Boswellia sacra* Flueck. oleoresin extracts from the Dhofar region of Oman**

Luay Rashan,^a^ Alan White,^b^ Manon Haulet,^b,c^ Nicolas Favelin,^b,c^ Parag Das,^d^ Ian Edwin Cock,^b,e*^

^a^Frankincense and Biodiversity Group, Dhofar University, Salalah 2509, Oman

^b^School of Environment and Science, Griffith University, 170 Kessels Rd, Nathan, Queensland 4111, Australia

^c^School of Biology, Ecole de Biologie Industrielle (EBI), Cergy, France

^d^Oman Pharmaceutical Products Co.LLC. Salalah/ Oman

^e^Environmental Futures Research Institute, Nathan Campus, Griffith University, 170 Kessels Rd, Nathan, Queensland 4111, Australia

*Corresponding author. Tel.: +61 7 37357637; fax: +61 7 37355282.

E-mail address: [I.Cock@griffith.edu.au](mailto:I.Cock@griffith.edu.au) (I. E. Cock).

**Supplementary Table 1:** Compounds putatively identified in *B. sacra* oleoresins by GC-MS analysis, their empirical formaula and a comparison of the calculated and measured molecular masses.

| **Compound Identification** | **Retention Time (min)** | **Empirical Formula** | **Calculated Molecular Mass** | **Measured Molecular Mass** |
| --- | --- | --- | --- | --- |
| 2,4(10)-Thujadiene | 12.501 | C10 H14 | 134.218 | 134.31 |
| p-Cymene | 15.26 | C10 H14 | 134.222 | 133.96 |
| Limonene | 15.497 | C10 H16 | 136.238 | 136.28 |
| γ-Terpineol | 15.94 | C10 H18 O | 154.253 | 154.33 |
| α-Pinene | 16.436 | C10 H16 | 136.238 | 136.17 |
| p-Cymenene | 17.454 | C10 H12 | 132.202 | 132.29 |
| Linalool | 17.764 | C10 H18 O | 154.253 | 154.33 |
| Thujone | 18.368 | C10 H16 O | 152.237 | 152.25 |
| Verbenol | 18.672 | C10 H16 O | 152.237 | 152.24 |
| (+)-Sabinol | 19.132 | C10 H16 O | 152.237 | 152.24 |
| p-Cymen-8-ol | 19.433 | C10 H14 O | 150.22 | 150.23 |
| Pinocarvone | 19.885 | C10 H14 O | 150.22 | 150.22 |
| α-Terpineol | 20.002 | C10 H18 O | 154.253 | 154.28 |
| Verbenone | 20.186 | C10 H14 O | 150.22 | 150.24 |
| Terpinen-4-ol | 20.361 | C10 H18 O | 154.253 | 154.33 |
| p-Cymen-8-ol | 20.559 | C12 H16 O2 | 192.25 | 192.27 |
| α-Terpineol | 20.768 | C10 H18 O | 154.253 | 154.29 |
| Sabinol | 21.144 | C10 H16 O | 152.237 | 151.87 |
| β-Elemene | 21.407 | C15 H24 | 204.357 | 204.42 |
| Carveol | 21.615 | C10 H16 O | 152.237 | 152.19 |
| Carvacrol | 22.392 | C10 H14 O | 150.22 | 150.26 |
| (-)-Bornyl acetate | 23.672 | C10 H20 O2 | 196.29 | 196.34 |
| Thymol | 24.022 | C10 H14 O | 150.22 | 150.23 |
| Caryophyllene | 28.572 | C15 H24 | 204.36 | 204.28 |
| Humulene | 30.016 | C15 H24 | 204.36 | 204.52 |
| γ-Muurolene | 30.75 | C15 H24 | 204.357 | 204.52 |
| Epicubenol | 34.623 | C15 H26 O | 222.366 | 222.19 |
| τ-Cadinol | 34.856 | C15 H26 O | 222.366 | 222.29 |
